# KDM6A Deficiency Induces Myeloid Bias and Promotes CMML‐Like Disease Through JAK/STAT3 Activation by Repressing SOCS3

**DOI:** 10.1002/advs.202413091

**Published:** 2025-05-14

**Authors:** Huiqiao Chen, Shufen Wang, Ruoyu Dong, Pinhui Yu, Tianyu Li, Liangning Hu, Mowang Wang, Zijun Qian, Hongyu Zhou, Xiaoyan Yue, Limengmeng Wang, Haowen Xiao

**Affiliations:** ^1^ Department of Hematology and Cell Therapy Sir Run Run Shaw Hospital Zhejiang University School of Medicine Hangzhou Zhejiang Province 310016 P. R. China; ^2^ Bone Marrow Transplantation Center The First Affiliated Hospital Zhejiang University School of Medicine Hangzhou Zhejiang Province 310009 P. R. China; ^3^ Institute of Hematology Zhejiang University Hangzhou Zhejiang Province 310058 P. R. China

**Keywords:** hematopoietic stem and progenitor cells, KDM6A, myelopoiesis, SOCS3, SYK

## Abstract

Chronic myelomonocytic leukemia (CMML) is a hematologic malignancy with a poor prognosis and limited targeted therapies. Lysine demethylase 6A (KDM6A), a H3K27 demethylase and key component of the COMPASS complex, is frequently mutated in hematologic malignancies, but its roles in embryonic hematopoiesis and tumor suppression in CMML remain unclear. Using zebrafish models with kdm6a mutants and integrative multi‐omics analysis (ATAC‐seq, RNA‐seq, ChIP), we find that Kdm6a is a critical positive regulator of hematopoietic stem and progenitor cell (HSPC) emergence via Syk‐related inflammatory signaling in a H3K27me3‐dependent manner. We further find that Kdm6a haploinsufficiency in zebrafish leads to myeloid‐biased hematopoiesis and a CMML‐like disease, similar to CMML patients with reduced KDM6A expression. This KDM6A haploinsufficiency also significantly alters the chromatin landscape of genes associated with aging and cellular homeostasis in HSPCs. Mechanistically, KAM6A haploinsufficiency represses SOCS3 expression, thereby activating JAK/STAT3 signaling in HSPCs. Importantly, inhibitors targeting JAK or STAT3 phosphorylation alleviate myeloid expansion, providing a rationale for JAK/STAT pathway inhibition in CMML therapy. These findings enhance our understanding of CMML pathogenesis and propose new therapeutic avenues.

## Graphical abstract

Lysine demethylase 6A (KDM6A), an epigenetic regulator expressed in hematopoietic stem and progenitor cells (HSPCs), plays a critical role in hematopoietic homeostasis. Deficiency of KDM6A induces myeloid lineage skewing and drives the development of a chronic myelomonocytic leukemia (CMML)‐like disease. Mechanistically, KDM6A promotes *SOCS3* transcription through chromatin accessibility regulation, which subsequently suppresses JAK/STAT3 signaling and downregulates myeloid‐associated gene expression.

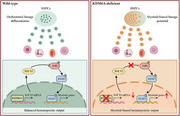

## 1 Introduction

Chronic myelomonocytic leukemia (CMML) is a prototypical chronic myeloid malignancy characterized by sustained peripheral blood monocytosis (absolute monocyte count ≥0.5 × 10^9/L, with monocytes accounting for ≥10% of the leukocyte count).^[^ ^1^ ^]^ CMML exhibits overlapping features of myeloproliferative neoplasms (MPN) and myelodysplastic syndromes (MDS), with an inherent risk of transformation to acute myeloid leukemia (AML).^[^ ^1^, ^2^, ^3^, ^4^ ^]^ Despite its distinct clinical features, the underlying pathogenesis of CMML remains largely unknown, thereby limiting the development of effective therapeutic options. Hypomethylating agents may temporarily improve clinical manifestations in responding patients but fail to diminish the impact of genomic alterations, thereby being unable to prevent the natural progression to acute leukemia.^[^ ^5^, ^6^, ^7^ ^]^ To date, allogeneic hematopoietic stem cell transplantation (allo‐HSCT) is the only potentially curative therapy for CMML. However, due to advanced age and prevalent comorbidities, the majority of CMML patients are not eligible for allo‐HSCT.^[^ ^1^, ^8^ ^]^ Therefore, there is an urgent need to explore optimal therapies for patients with CMML.

Lysine demethylase 6A (KDM6A), also known as ubiquitously transcribed tetratricopeptide repeat protein on chromosome X (UTX), is an X‐linked histone demethylase expressed in hematopoietic stem and progenitor cells (HSPCs). KDM6A has garnered significant attention in cancer research due to its dual roles as being an oncogene or a tumor suppressor.^[^ ^9^, ^10^, ^11^, ^12^, ^13^ ^]^ Importantly, mechanistic insights into the role of KDM6A in malignancies are multifaceted and involve complex regulatory networks. KDM6A antagonizes the polycomb repressive complex 2 (PRC2)‐mediated H3K27me3, which is catalyzed by the methyltransferase enhancer of zeste homologue 2 (EZH2), thereby regulating H3K27me3 levels and affecting multiple biological processes.^[^ ^14^, ^15^ ^]^ Additionally, KDM6A interacts with other epigenetic modifiers, such as lysine methyltransferase 2C (KMT2C) and lysine methyltransferase 2D (KMT2D), leading to alterations in H3K27 acetylation, H3K4 monomethylation, and chromatin accessibility at enhancers, independent of its demethylase function.^[^ ^15^, ^16^, ^17^, ^18^, ^19^, ^20^ ^]^ KDM6A also regulates diverse immune cell functions. For instance, during infection of chronic lymphocytic choriomeningitis virus, KDM6A enhances CD8^+^ T cell‐mediated antiviral responses independently of H3K27me3 demethylation.^[^ ^21^ ^]^ In thymocyte development, KDM6A is essential for the differentiation of CD4^+^ T cells from CD4^+^CD8^+^ thymic precursors.^[^ ^22^ ^]^ Notably, emerging evidence identifies KDM6A as a critical regulator of sex‐specific differences in natural killer (NK) cells, where it governs both cellular fitness and effector functions in a dose‐dependent manner.^[^ ^23^ ^]^

Deletions or mutations of the *KDM6A* gene have been identified in 8% of CMML patients, a rate significantly higher than that of 2% found in AML patients.^[^ ^24^, ^25^ ^]^ These mutations identified in hematologic malignancies are usually frameshift or nonsense, which are located in proximity to the tetratricopeptide repeats (TRPs) domain or the Jumonji C (JmjC) domain, resulting in C‐terminal truncation and loss of the JmjC domain.^[^ ^25^, ^26^ ^]^ Given the presence of lysine demethylase 6c (Kdm6c) on the Y chromosome, Kdm6a‐null male embryos are capable of surviving to adulthood, while loss of Kdm6a is embryonically lethal in homozygous knockout females.^[^ ^27^ ^]^ Thus, the explicit roles of KDM6A in hematopoiesis, particularly during embryonic stages, remain poorly understood. Furthermore, since *KDM6A* can escape X chromosome inactivation,^[^ ^28^, ^29^ ^]^ female patients usually exhibit KDM6A haploinsufficiency when one allelic gene is mutated. To date, the involvement of KDM6A haploinsufficiency in hematologic disorders remains largely uncharacterized.

The zebrafish (Danio rerio, Dr) genome and hematopoietic system closely resemble those in humans, serving as an excellent model to study hematopoiesis, particularly during early embryogenesis.^[^ ^30^ ^]^ Herein, we established zebrafish models with a series of *kdm6a* mutants to investigate the functions of *kdm6a* during HSPC emergence and differentiation, and to explore the underlying molecular mechanisms. We identified Kdm6a as a positive regulator of HSPC emergence, mediated by spleen tyrosine kinase (Syk)‐related inflammatory signaling in a H3K27me3‐dependent manner. Of note, we also found that Kdm6a haploinsufficiency induced skewed myeloid differentiation and progression to CMML‐like disease in zebrafish, in which deregulation of suppressor of cytokine signaling 3a (Socs3a) and aberrant activation of Jak/Stat3 signaling were involved. Treatment with the inhibitors targeting Jak/Stat3 could effectively inhibit the expansion of monocytes in vivo.

## 2 Results

### 2.1 Generation of Zebrafish Mutants Carrying *kdm6a* C‐Terminal Truncation

Mammalian KDM6A and zebrafish Kdm6a proteins are evolutionarily conserved. The zebrafish Kdm6a shares ≈75% amino acid identity with human KDM6A.^[^ ^31^ ^]^ They share a highly conserved key architecture, including TPRs and JmjC domains (Figure S1A,B, Supporting Information), suggesting that the in vivo roles of Kdm6a in zebrafish hematopoiesis can provide valuable insights into the functions of human KDM6A. To determine whether Kdm6a is involved in hematopoiesis, CRISPR/Cas9 methods were used to target the exon10 or the exon23 of the zebrafish *kdm6a* gene (Figure S2A, Supporting Information). Two independent *kdm6a* mutants were generated, with 10 bp deletions in exon10 of *kdm6a* ^e10 (△10)^ (Figure S2B, Supporting Information), and 1 bp deletion in exon23 of *kdm6a* ^e23 (△1)^ (Figure S2C, Supporting Information). Both mutations were predicted to result in premature stop codons leading to the loss of Kdm6a (Figure S2B,C, Supporting Information), similar to mutations identified in myeloid malignancies. The zebrafish *kdm6a*‐null homozygous embryos, either *kdm6a* ^e10 (△10/ △10)^ or *kdm6a* ^e23 (△1/ △1)^, did not show a lower survival rate compared to their wild‐type (WT) counterparts during early embryogenesis (Figure S2D,E, left panels, Supporting Information). However, adult zebrafish *kdm6a*‐null homozygous mutants (*kdm6a* ^e10 (△10/ △10)^ or *kdm6a* ^e23 (△1/ △1)^) exhibited significantly lower survival rates compared with their WT siblings or heterozygous mutants (Figure S2D,E, right panels, Supporting Information). Almost 80%∼ of the surviving *kdm6a*‐null homozygous adults exhibited dwarfism and reduced body weight (data not shown). Furthermore, during follow‐up at 12–13 months, the survival rates of *kdm6a*‐null homozygous mutants gradually declined with age, and even heterozygous mutants (*kdm6a* ^e10 (+/△10)^ or *kdm6a* ^e23 (+/△1)^) exhibited a trend of reduced survival compared with control WT counterparts (Figure S2F,G, Supporting Information).

The two mutations led to loss of Kdm6a and exhibited similar hematological phenotypes. Therefore, unless specifically mentioned, we used *kdm6a* ^e23 (△1)^ in downstream analyses. Zebrafish *kdm6a*‐null homozygous mutant is referred to as *kdm6a* ^−/−^, whereas the *kdm6a* haploinsufficiency mutant is referred to as *kdm6a* ^+/−^.

### 2.2 Kdm6a is Important for HSPC Production During Early Development

**Figure 1:**
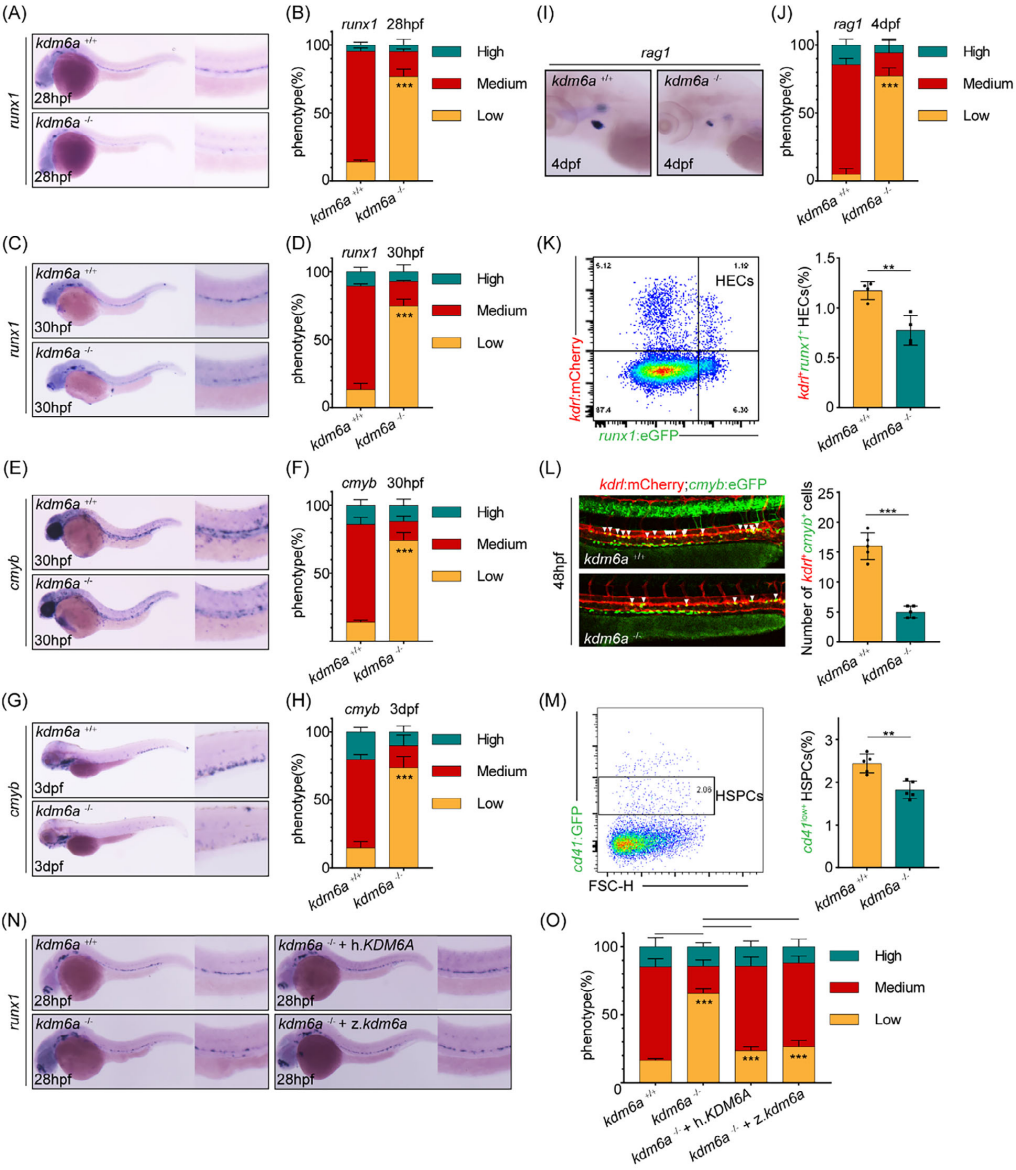
*kdm6a* is required for HSPC emergence in zebrafish. A,B) WISH for *runx1* A) and %phenotype distribution B) in embryos at 28 hpf as indicated (n = 4, mean ± SD, Student's t test). C,D) WISH for *runx1* C) and %phenotype distribution D) in embryos at 30 hpf as indicated (n = 4, mean ± SD, Student's t test). E,F) WISH for *cmyb* E) and %phenotype distribution F) in embryos at 30 hpf as indicated (*n* = 4, mean ± SD, Student's t test). G,H) WISH for *cmyb* G) and %phenotype distribution H) in embryos at 3 dpf as indicated (n = 4, mean ± SD, Student's t test). I,J) WISH for *rag1* I) and %phenotype distribution J) in embryos at 4 dpf as indicated (n = 4, mean ± SD, Student's t test). K) Flow cytometry plots of *kdrl*:mCherry^+^; *runx1*:eGFP^+^ double positive cells at 28 hpf (left). Graphs depicting the percentage of *kdrl*:mCherry^+^; *runx1*:eGFP^+^ hemogenic endothelial cells per embryo at 28 hpf (right) (*n* = 4, mean ± SD, Student's t test). L) Confocal imaging of *kdrl*:mCherry^+^; *cmyb*:eGFP^+^ hemogenic endothelial cells in AGM at 48 hpf (left, white arrowheads). Graphs depicting the number of *kdrl*:mCherry^+^; *cmyb*:eGFP^+^ cells per embryo at 48 hpf (right) (*n* = 5, mean ± SD, Student's t test). M) Flow cytometry plots of *cd41*:eGFP^low^ cells at 3 dpf (left). Graphs depicting the percentage of *cd41*:eGFP^low^ HSPCs per embryo at 3 dpf (right) (*n* = 5, mean ± SD, Student's t test). N,O) WISH for *runx1* N) and %phenotype distribution O) in embryos at 28 hpf as indicated (*n* = 4, mean ± SD, one‐way ANOVA). **p <0.01; ***p < 0.001.

To investigate whether Kdm6a regulates embryonic hematopoiesis in zebrafish, we focused on its potential roles in definitive hematopoiesis. Whole‐mount in situ hybridization (WISH) of *kdm6a* ^−/−^ embryos showed a reduced expression of *runx1*, a marker for hemogenic endothelial cells (HECs) and HSPCs, at 28‐ and 30 h post‐fertilization (hpf), compared with their WT siblings (**Figure** **1**A–D). This impaired HSPC production was further confirmed using another HSPC‐specific marker, *cmyb*, at 30 hpf. (Figure 1E,F). The *kdm6a* ^−/−^ embryos also exhibited reduced expression of *cmyb* in their caudal hematopoietic tissue (CHT) at 3 days post‐fertilization (dpf), and of *rag1* in their thymus at 4 dpf, indicating that the phenotype of impaired HSPC production persisted throughout embryonic development (Figure 1G–J). After crossing *kdm6a* mutants with the Tg(*runx1*:eGFP/*kdrl*:mCherry) reporter line, flow cytometry analysis revealed a significant decrease in the number of double‐positive (*kdrl^+^runx1^+^*) HECs in *kdm6a* ^−/−^ embryos (Figure 1K). To further confirm the necessity of Kdm6a for HSPC generation, we assessed live definitive hematopoietic precursors using the Tg(*kdrl*:mCherry/*cmyb*:eGFP) double‐transgenic line. The number of *kdrl* and *cmyb* double‐positive precursors in the ventral wall of the dorsal aorta (VDA) region was markedly reduced in the *kdm6a* ^−/−^ embryos (Figure 1L). Additionally, the well‐established HSPC transgenic line Tg(*cd41*:eGFP) indicated a dramatic reduction in GFP^low^ HSPCs in the CHT at 3 dpf in *kdm6a* ^−/−^ embryos (Figure 1M). To further characterize the phenotypes caused by Kdm6a loss‐of‐function, we used a *kdm6a* translation‐blocking morpholino (MO), which presented significantly reduced Kdm6a protein levels (Figure S3A, Supporting Information). WISH analysis demonstrated impaired HSPC production in kdm6a morphants, evidenced by decreased *runx1* expression in the VDA region and decreased *cmyb* expression in the CHT region (Figure S3B–E, Supporting Information). The population of lymphoid cells labeled by *rag2* and *coro1a* was also significantly decreased in *kdm6a* morphants (Figure S3F, Supporting Information). Using quantitative real time polymerase chain reaction (qPCR) analysis, decreased *runx1* and *cmyb* expression levels were identified in whole embryos, which is consistent with data obtained from WISH and further suggested impaired HSPC emergence (Figure S3G, Supporting Information).

Considering that the earliest HSPCs originate from the HECs in the VDA, the aforementioned HSPC deficiency in *kdm6a* ^−/−^ embryos may be attributed to disruption in vessel formation or arterial specification. To exclude this possibility, we examined the expression of arterial endothelial markers *kdrl*, *dlc* and *tbx20* in *kdm6a* ^−/−^ embryos. The expression levels of these markers were not changed (Figure S4A–C, Supporting Information). Consistently, using Tg(*fli1a*:eGFP) at 28 hpf by confocal microscopy, no alterations was observed between *kdm6a* ^−/−^ and control groups (Figure S4D, Supporting Information), indicating normal vessel development and artery‐vein establishment.

### 2.3 Kdm6a Controls HSPC Generation by Regulating Inflammatory Pathways in a Demethylase‐Dependent Manner

**Figure 2:**
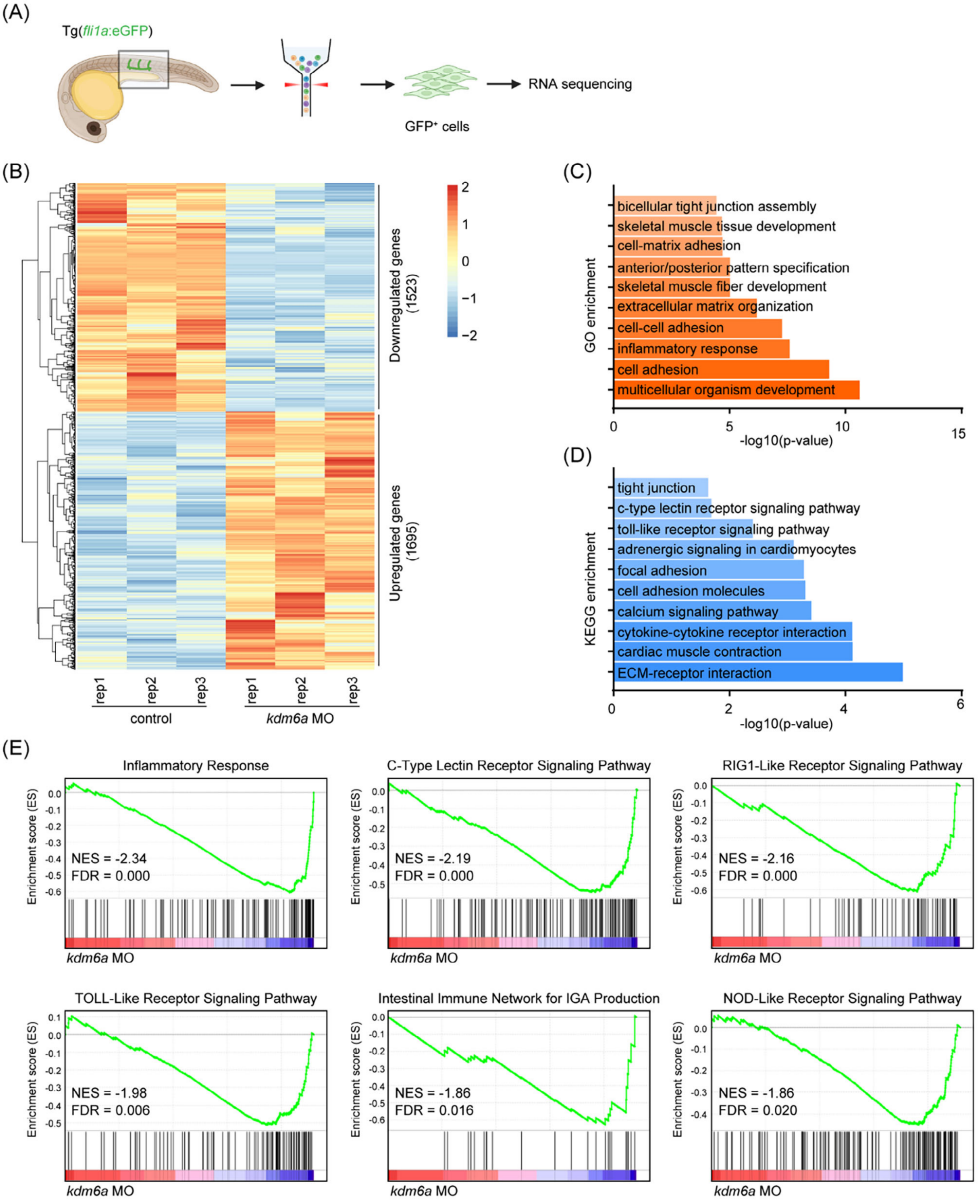
Loss of Kdm6a deregulates inflammatory gene expression in ECs. A) Flowchart of sorting and RNA sequencing. B) Heatmap of differentially expressed genes in endothelial cells from *kdm6a* morphants and their WT controls at 28 hpf via RNA‐seq (cut‐off fold change 1.5, *p* < 0.05). C) Gene ontology (GO) enrichment analysis of biological processes based on differentially expressed genes identified through RNA‐seq. D) Kyoto Encyclopedia of Genes and Genomes (KEGG) analysis based on differentially expressed genes identified through RNA‐seq. E) Gene set enrichment analysis (GESA) of RNA expression profiles in endothelial cells from *kdm6a* morphants and their WT controls at 28 hpf.

To date, no study has reported the involvement of Kdm6a in HSPC generation. As such we further investigated its underlying mechanisms. Both *kdm6a* translation‐blocking morphants and *kdm6a* ^−/−^ mutants exhibited a similar phenotype of HSPC deficiency as described above. We subsequently used *kdm6a* translation‐blocking morphants to understand how the absence of Kdm6a affects HSPC generation. As shown in **Figure** **2**A, endothelial cells (ECs) labeled with *fli1a*:eGFP were isolated from *kdm6a* morphants and their WT controls at 28 hpf for RNA‐sequencing (RNA‐seq) analysis. A total of 1695 genes were significantly upregulated, and 1523 genes were significantly downregulated (fold change 1.5, *p* < 0.05) in ECs from *kdm6a* morphants compared with controls (Figure 2B). Gene Ontology (GO) analysis indicated a significant enrichment of inflammatory response‐related genes among differentially expressed genes (DEGs) in *kdm6a* morphants (Figure 2C). Kyoto Encyclopedia of Genes and Genomes (KEGG) analysis also revealed strong enrichment of terms related to “cytokine‐cytokine receptor interaction”, “toll‐like receptor signaling pathway”, and “c‐type lectin receptor signaling pathway” (Figure 2D). Consistently, gene set enrichment analysis (GSEA) showed that many significantly downregulated hallmark genes in *kdm6a* morphants are associated with inflammatory response, including “c‐type lectin receptor signaling pathway”, “rig‐1‐like receptor signaling pathway”, “toll‐like receptor signaling pathway”, “intestinal immune network for IGA production”, and “nod‐like receptor signaling pathway” (Figure 2E).

**Figure 3:**
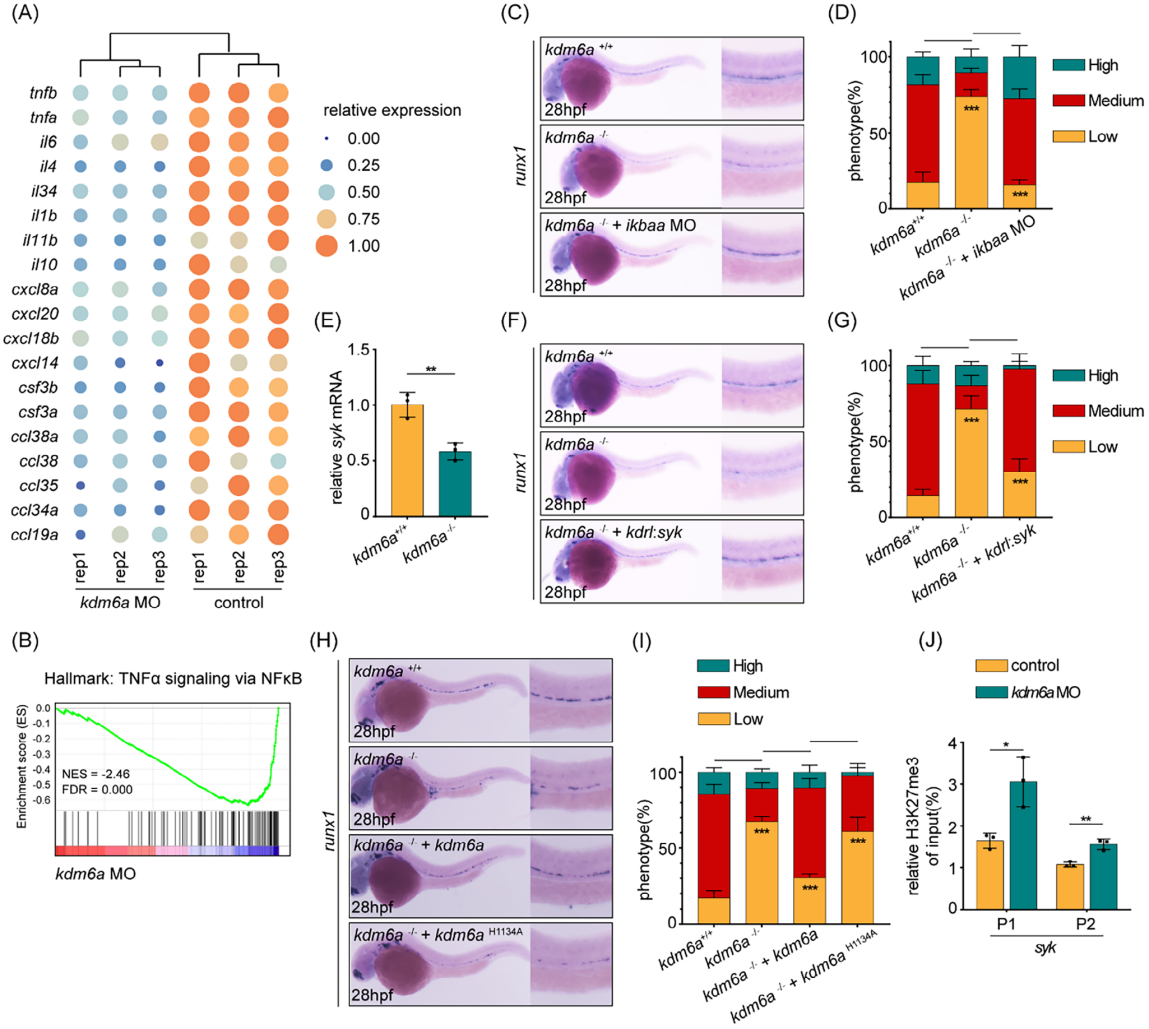
Kdm6a regulates HSPC production via Syk‐associated inflammatory response in a H3K27me3‐dependent manner. A) Heatmap showing differential expression of inflammatory‐associated genes. B) Enrichment plot of the TNFα signaling via NF‐κB between differentially regulated genes in *kdm6a* morphants and their WT controls by GSEA. C,D) WISH for *runx1* C) and %phenotype distribution D) in WT control, *kdm6a* mutants, and *kdm6a* mutants with *ikbaa* morphants at 28 hpf as indicated (*n* = 4, mean ± SD, one‐way ANOVA). E) qPCR analysis of *syk* expression in sorted endothelial cells from WT control and *kdm6a* mutants at 28 hpf (n = 3, mean ± SD, Student's t test). F,G) WISH for *runx1* F) and %phenotype distribution G) in WT control, *kdm6a* mutants, and *kdm6a* mutants with *kdrl*:*syk* constructs at 28 hpf (*n* = 4, mean ± SD, one‐way ANOVA). H,I) WISH for *runx1* H) and %phenotype distribution I) in WT control, *kdm6a* mutants, *kdm6a* mutants with *kdm6a* mRNA, and *kdm6a* mutants with *kdm6a* ^H1134A^ mRNA at 28 hpf (*n* = 4, mean ± SD, one‐way ANOVA). J) ChIP‐qPCR analyses of *syk* promoter in *kdm6a* morphants and their WT controls by using an anti‐H3K27me3 antibody at 28 hpf (n = 3, mean ± SD, Student's t test). *p <0.05; **p <0.01; ***p < 0.001.

It has been well established that the activation of inflammatory pathways is necessary for HSPC emergence. Proinflammatory factors, including interleukin (IL)‐1, IL‐6, and tumor necrosis factor α (TNFα), are involved in the development of embryonic HSPCs in the VDA region.^[^ ^32^, ^33^, ^34^, ^35^ ^]^ RNA‐seq data in our study showed that the expression levels of inflammation‐related genes, including TNF family, chemokines, and cytokines, were downregulated in *kdm6a* morphants (**Figure** **3**A). GSEA analysis further demonstrated a significant correlation between down‐regulated nuclear factor kappa B (NF‐κB) transcriptional programs and genes induced by Kdm6a loss (Figure 3B). We further investigated whether Kdm6a regulates HSPC generation through the NF‐κB signaling pathway. Knockdown the gene of inhibitor of kappa B alpha a (IκBaa), which encodes an inhibitory protein that binds to NF‐κB and prevents its translocation to the nucleus, effectively restored the population of HSPCs in *kdm6a* ^−/−^ embryos (Figure 3C,D). These data support that Kdm6a regulates NF‐κB signaling in numerous cell types, including HSPCs and endothelial cells (ECs).

NF‐κB has been identified as a central mediator of the inflammatory response, with numerous downstream targets and upstream inducers.^[^ ^36^, ^37^ ^]^ The signaling pathway of C‐type lectin receptors through Syk is a well‐known activator of NF‐κB.^[^ ^38^, ^39^, ^40^ ^]^ Consistently, our data indicated by qPCR analysis that ECs from the trunk region of *kdm6a* ^−/−^ embryos exhibited reduced *syk* expression (Figure 3E), suggesting that *syk* is a potential downstream target of Kdm6a. To determine whether impaired HSPC generation was associated with decreased *syk* expression, Syk was depleted in WT embryos using CRISPR/Cas9 with *syk* sgRNA and Cas9 protein (Figure S5A, Supporting Information). Embryos with *syk* depletion exhibited a significantly decreased number of *runx1* ^+^ cells and *cmyb* ^+^ cells compared with WT embryos (Figure S5B,C, Supporting Information). Subsequently, a construct expressing *syk* driven by the *kdrl* promoter was generated. As expected, ectopic expression of *syk* in ECs rescued HSPC formation in *kdm6a* ^−/−^ embryos (Figure 3F,G). Collectively, our data suggest that Kdm6a acts as a positive regulator of HSPC emergence, which is mediated through a Syk‐dependent inflammatory response.

The mechanisms of Kdm6a‐induced gene activation have been primarily attributed to its demethylase catalytic function. To determine whether the demethylase activity of Kdm6a is essential for HSPC generation, a catalytically inactive Kdm6a mutant, H1134A, was expressed in *kdm6a* ^−/−^ embryos. Notably, the ectopic expression of Kdm6a H1134A mutant in *kdm6a* ^−/−^ embryos did not exhibit the similar restorative effect on HSPC deficiency as expression of Kdm6a WT in *kdm6a* ^−/−^ embryos (Figure 3H,I). More importantly, loss of Kdm6a led to a significant increase in the level of H3K27me3 in the promoter region of *syk* gene, as measured by chromatin immunoprecipitation coupled with quantitative PCR (ChIP‐qPCR) (Figure 3J). Taken together, these findings indicate that Kdm6a controls HSPC generation by regulating *syk* expression in a H3K27me3‐dependent manner through its demethylase activity.

### 2.4 Haploinsufficiency of Kdm6a Induced Skewed Myeloid Differentiation and Progression to a CMML‐Like Disease in Zebrafish

**Figure 4:**
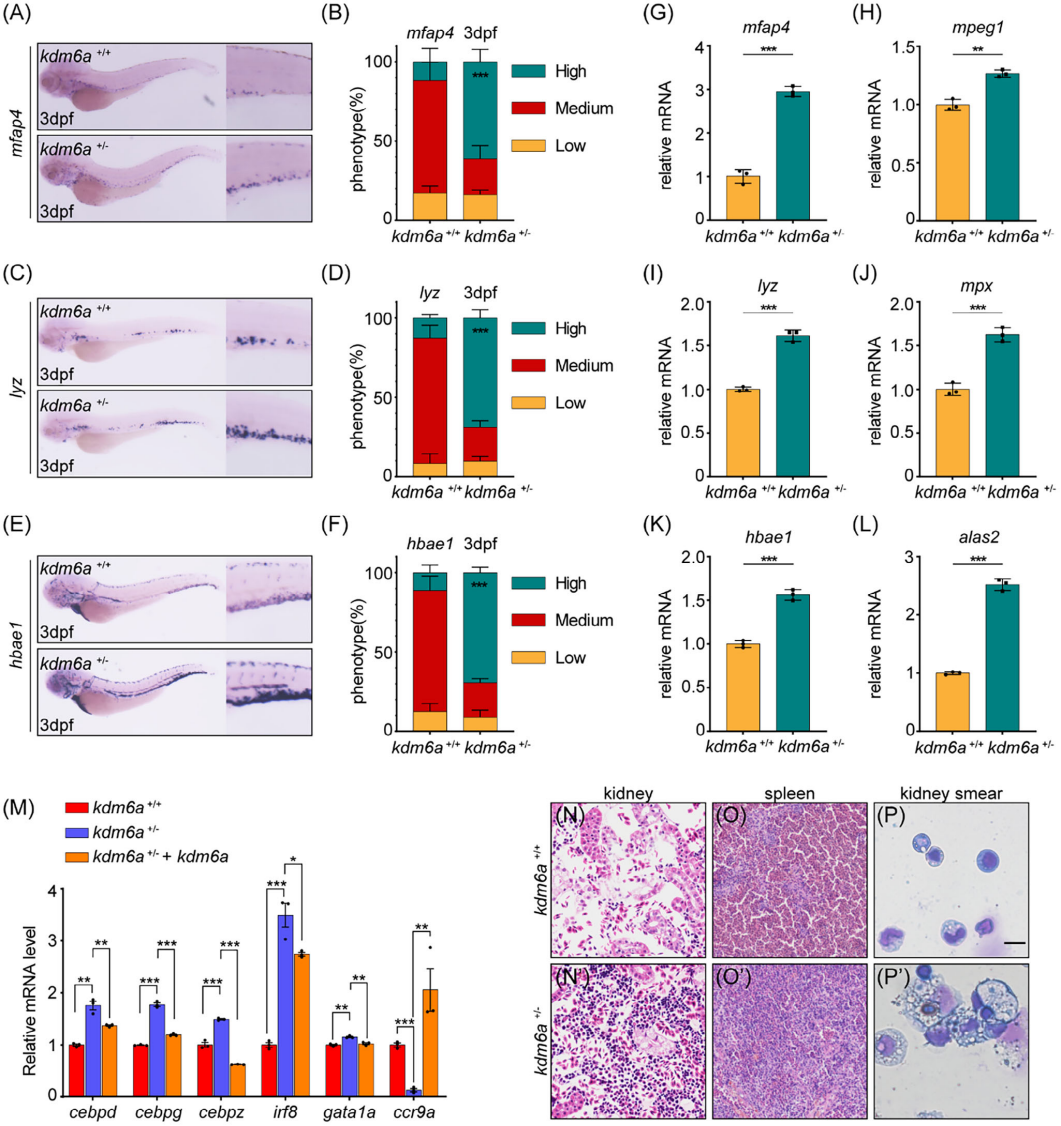
Haploinsufficiency of Kdm6a has skewed myelopoiesis in embryonic and adult zebrafish. A,B) WISH for *mfap4* A) and %phenotype distribution B) in embryos at 3 dpf as indicated (n = 4, mean ± SD, Student's t test). C,D) WISH for *lyz* C) and %phenotype distribution D) in embryos at 3 dpf as indicated (*n*  = 4, mean ± SD, Student's t test). E,F) WISH for *hbae1* E) and %phenotype distribution F) in embryos at 3 dpf as indicated (n = 4, mean ± SD, Student's t test). G–L) qPCR analysis of *mfap4*, *mpeg1*, *lyz*, *mpx*, *hbae1*, and *alas2* expression in WT control and *kdm6a* ^+/−^ mutant embryos at 3 dpf (n = 3, mean ± SD, Student's t test). M) qPCR analysis of lineage differentiation‐related transcription factors expression including *cebpd*, *cebpg*, *cebpz*, *irf8*, *gata1a*, and *ccr9a* in *cd41*:eGFP^low^ HSPCs isolated from WT control or *kdm6a* ^+/−^ mutant embryos (n = 3, mean ± SD, one‐way ANOVA). N‐N’) Hematoxylin and eosin staining of paraffin‐embedded sections of kidney from representative WT control or *kdm6a* ^+/−^ mutant adults. O‐O’) Hematoxylin and eosin staining of paraffin‐embedded sections of spleen from representative WT control or *kdm6a* ^+/−^ mutant adults. P‐P’) May–Grünwald–Giemsa staining of whole kidney marrow (KM) cells presentative 3 *kdm6a* ^+/−^ fish with CMML‐like phenotypes (totally 12 *kdm6a* ^+/−^ adults were used for experiment). *p <0.05; **p <0.01; ***p < 0.001.

*KDM6A* is a pseudoautosomal gene that can escape X inactivation and frequently exhibits haploinsufficient expression in hematologic malignancies when mutated.^[^ ^13^, ^23^, ^41^ ^]^ Previous studies have found that KDM6A haploinsufficiency regulates the numbers and effector functions of lymphoid cells.^[^ ^14^, ^23^ ^]^ However, the dosage effect of KDM6A on myelopoiesis remains unclear. To explore the impact of Kdm6a haploinsufficiency on myeloid differentiation in vivo, we analyzed a series of lineage markers in *kdm6a* ^+/−^ embryos. WISH analysis showed a significant increase in the expression level of the macrophage marker *mfap4* in *kdm6a* ^+/−^ embryos, compared with *kdm6a* ^+/+^ siblings at 72 hpf (**Figure** **4**A,B). Additionally, the expression levels of the neutrophil marker *lyz* and the erythroid marker *hbae1* were also up‐regulated in the *kdm6a* ^+/−^ CHT (Figure 4C–F). The increased mRNA levels of macrophage genes, *mfap4* and *mpeg1*, were confirmed by qPCR analysis in homogenized larvae (Figure 4G,H). Consistent with the WISH results, mRNA levels of neutrophil genes (*lyz*, *mp*x) and erythroid genes (*hbae1*, *alas2*) were also elevated (Figure 4I–L). These results indicate that *kdm6a* ^+/−^ mutants exhibit a bias toward myeloid lineage commitment. To investigate whether the expansion of myeloid cells in *kdm6a* ^+/−^ embryos may originate from those HSPCs with an enhanced capability of myeloid differentiation, *cd41*:eGFP^low^ HSPCs were sorted from *kdm6a* ^+/+^ and *kdm6a* ^+/−^ CHT, respectively, followed by qPCR analysis. Expression levels of the master regulators of myeloid differentiation in *kdm6a* ^+/−^ HSPCs, such as *c/ebp* factors, *irf8*, and *gata1a*, were higher than those in *kdm6a* ^+/+^ siblings. While the expression level of *ccr9a*, an essential regulator of lymphoid differentiation, was lower (Figure 4M).

Based on myeloid bias observed in *kdm6a* ^+/−^ mutants during early development, we further explored whether this skewed myelopoiesis persisted in adult hematopoiesis. At the age of 18 months, hematoxylin and eosin (H&E) staining was performed on multiple tissue sections. The *kdm6a* ^+/+^ zebrafish kidney is composed of nephron functional units in arborized arrangements, surrounded by hematopoietic tissue that is dispersed throughout the intervening spaces. Instead, *kdm6a* ^+/−^ zebrafish exhibited infiltration and accumulation of hematopoietic cells throughout these spaces (Figure 4N). Moreover, the *kdm6a* ^+/−^ zebrafish also exhibited abnormal infiltration of hematopoietic cells in spleen (Figure 4O). To identify the composition of these cells, May Grünwald‐Giemsa staining was performed on the whole kidney marrow (KM) cells. Pronounced monocytosis was particularly observed in 18‐month‐old *kdm6a* ^+/−^ mutants, simulating the characteristics of CMML patients (Figure 4P).

### 2.5 Haploinsufficiency of Kdm6a Represses *socs3a* Expression to Activate Jak/Stat3 Signaling in Zebrafish

**Figure 5:**
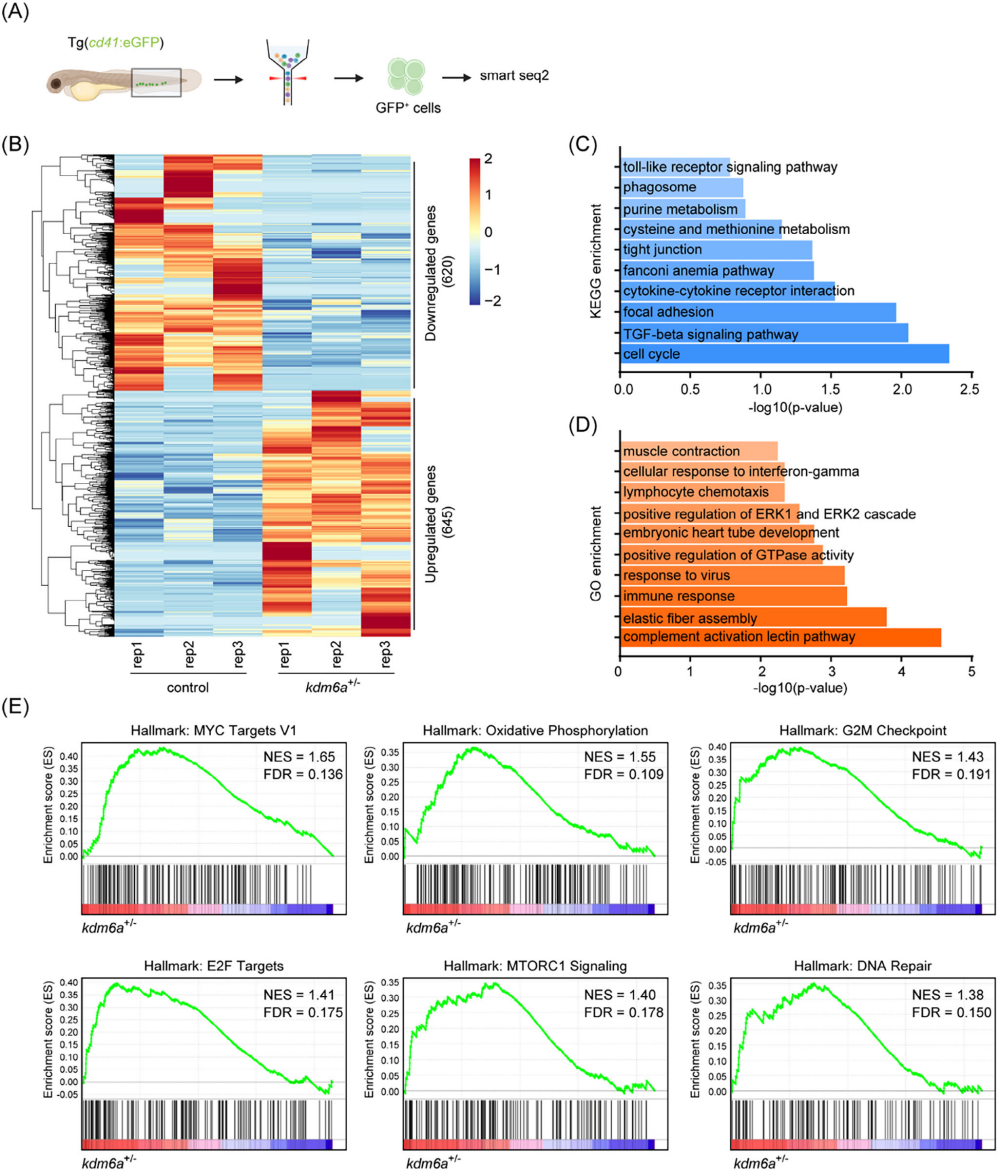
Haploinsufficiency of Kdm6a dysregulates aging related gene expression in HSPCs. A) Flowchart of sorting and RNA sequencing. B) Heatmap of differentially expressed genes in HSPCs from *kdm6a* ^+/−^ mutants and their WT controls at 3 dpf via RNA‐seq (cut‐off fold change 1.5, *p* < 0.05). C) Gene ontology (GO) enrichment analysis of biological processes based on differentially expressed genes identified through RNA‐seq. D) Kyoto Encyclopedia of Genes and Genomes (KEGG) analysis based on differentially expressed genes identified through RNA‐seq. E) Gene set enrichment analysis (GESA) of RNA expression profiles in HSPCs from *kdm6a* ^+/−^ mutants and their WT controls at 3 dpf.

**Figure 6:**
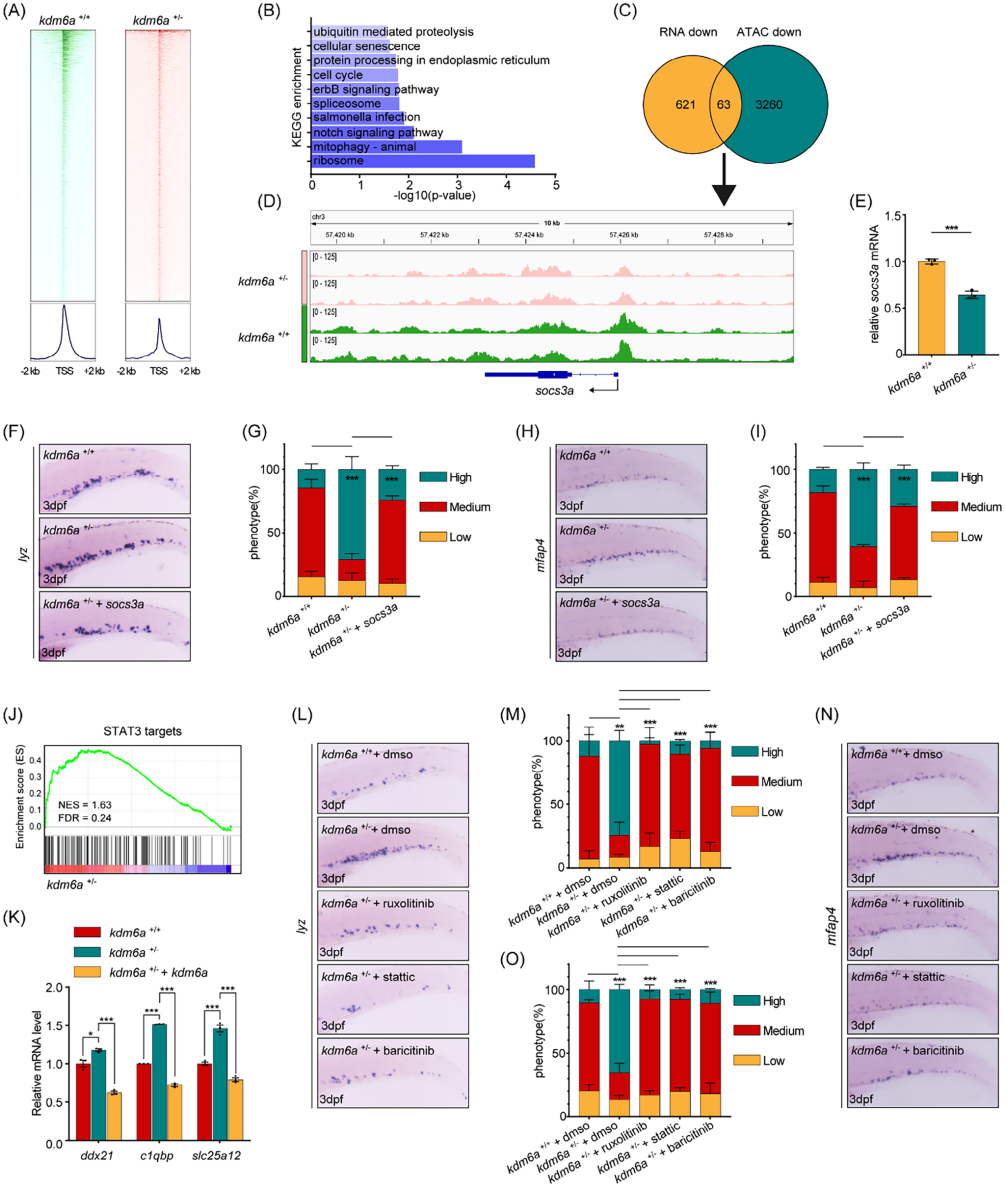
Haploinsufficiency of Kdm6a promotes myeloid‐biased hematopoiesis through repressing *socs3a* and activating Jak/Stat3 signaling. A) Heatmaps showing the binding signals of ATAC at the promoter regions (TSS±2kb) in *cd41*:eGFP^low^ HSPCs at 3 dpf from WT control and *kdm6a* ^+/−^ mutants. B) Kyoto Encyclopedia of Genes and Genomes (KEGG) analysis based on genes with differentially accessible chromatin at promoter regions. C) Venn plot showing the overlap of genes with specific accessible chromatin at promoter regions and downregulated expression in *kdm6a* ^+/−^ HSPCs. Genes for ATAC‐seq were assigned by differentially accessible chromatin at promoter regions. D) Genome browser tracks for ATAC enrichment signals across the *socs3a* locus in WT control and *kdm6a* ^+/−^ mutants. E) qPCR analysis of *socs3a* expression in sorted HSPCs from WT control and *kdm6a* ^+/−^ mutants at 3 dpf (n = 3, mean ± SD, Student's t test). F,G) WISH for *runx1* F) and %phenotype distribution G) in WT control, *kdm6a* ^+/−^ mutants, and *kdm6a* ^+/−^ mutants with *socs3a* mRNA at 3 dpf (*n* = 4, mean ± SD, one‐way ANOVA). (H‐I) WISH for *mfap4* H) and %phenotype distribution I) in WT control, *kdm6a* ^+/−^ mutants, and *kdm6a* ^+/−^ mutants with *socs3a* mRNA at 3 dpf (*n* = 4, mean ± SD, one‐way ANOVA). J) Enrichment plot of the STAT3 targets between differentially regulated genes in *kdm6a* ^+/−^ HSPCs and their WT controls by GSEA. K) qPCR analysis of STAT3 targets including *ddx21*, *c1qbp*, and *slc25a12* in *cd41*:eGFP^low^ HSPCs isolated from WT control and *kdm6a* ^+/−^ mutant embryos (n  = 3, mean ± SD, one‐way ANOVA). (L‐M) WISH for *lyz* L) and %phenotype distribution M) in WT control, *kdm6a* ^+/−^ mutants, *kdm6a* ^+/−^ mutants with ruxolitinib, *kdm6a* ^+/−^ mutants with static, and *kdm6a* ^+/−^ mutants with baricitinib at 3 dpf (*n* = 3, mean ± SD, one‐way ANOVA). N,O) WISH for *mfap4* N) and %phenotype distribution O) in WT control, *kdm6a* ^+/−^ mutants, *kdm6a* ^+/−^ mutants with ruxolitinib, *kdm6a* ^+/−^ mutants with static, and *kdm6a* ^+/−^ mutants with baricitinib at 3 dpf (n = 3, mean ± SD, one‐way ANOVA). *p <0.05; **p <0.01; ***p < 0.001.

To elucidate the mechanisms by which Kdm6a haploinsufficiency disrupts the diverse and balanced differentiation of HSPCs, RNA‐seq analysis was used to profile isolated HSPCs labeled with *cd41*:eGFP^low^ from *kdm6a* ^+/−^ and *kdm6a* ^+/+^ zebrafish at 3 dpf, respectively (**Figure** **5**A). A total of 645 genes were significantly upregulated, and 620 genes were significantly downregulated (fold change 1.5, p < 0.05) in *kdm6a* ^+/−^ HSPCs compared with WT controls (Figure 5B). KEGG analysis revealed an enrichment of gene sets associated with “cell cycle”, “TGF‐β signaling pathway”, “cysteine and methionine metabolism” and “purine metabolism” in DEGs (Figure 5C). GO analysis highlighted an enrichment of inflammatory signature genes in *kdm6a* ^+/−^ HSPCs, such as “complement activation lectin pathway”, “immune response”, and “lymphocyte chemotaxis” (Figure 5D). Notably, GSEA identified several significantly upregulated hallmark genes in *kdm6a* ^+/−^ HSPCs, including gene sets like “MYC Targets”, “Oxidative Phosphorylation”, “G2 M Checkpoint”, “E2F Targets” “MTORC1 Signaling”, and “DNA Repair” (Figure 5E), suggesting that haploinsufficiency of Kdm6a induces aging and homeostasis imbalance in HSPCs. Kdm6a facilitates gene activation through its demethylase‐dependent function of JmjC domain, as well as its demethylase‐independent role as a component of the COMPASS‐like complex. Next, we investigated whether haploinsufficiency of Kdm6a could alter chromatin accessibility. Transposase‐Accessible Chromatin sequencing (ATAC‐seq) was performed on *kdm6a* ^+/−^ and *kdm6a* ^+/+^ HSPCs, respectively. By analyzing ATAC‐seq peak signals at promoters (TSS±2kb), we found that the chromatin accessibility overall decreased in Kdm6a–haploinsufficient HSPCs (**Figure** **6**A). Specifically, chromatin accessibility at the promoters of 3260 genes decreased in *kdm6a* ^+/−^, while there was an increase in chromatin accessibility at the promoters of 361 genes. Genes with differentially accessible in promoter regions induced by Kdm6a haploinsufficiency were enriched in terms of “mitophagy”, “cell cycle”, “protein processing in endoplasmic reticulum”, “cellular senescence”, and “ubiquitin mediated proteolysis” (Figure 6B). Importantly, consistent to our RNA‐seq results, ATAC‐seq findings suggest that Kdm6a haploinsufficiency affects the chromatin accessibility of genes related to the aging and cellular homeostasis in HSPCs.

Then, we integrated data of RNA‐seq and ATAC‐seq, and found that there were 63 genes in *kdm6a* ^+/−^ HSPCs that simultaneously exhibited decreased chromatin accessibility at promoters and reduced transcriptional levels (Figure 6C). Among these genes, *socs3a* was notably altered and caught our attention (Figure 6D). The decreased transcription level of *socs3a*, confirmed by qPCR, indicated that *socs3a* is a direct downstream target of Kdm6a (Figure 6E). We then investigated whether the down‐regulation of *socs3a* contributes to the myeloid‐biased differentiation of *kdm6a* ^+/−^ HSPCs. We designed several sgRNAs and selected the one destroying the SH2 domain that most efficiently impaired Socs3a function (Figure S6A, Supporting Information). WT embryos injected with this *socs3a* sgRNA and Cas9 protein exhibited significantly higher numbers of *lyz* ^+^/*mpx* ^+^ neutrophils, *mfap4* ^+^ monocytes, and *hbae1* ^+^ erythrocytes than those in uninjected WT embryos (Figure S6B–E, Supporting Information), suggesting that Socs3a is involved in the regulation of myelopoiesis.

Then, we constructed ectopic expression of *socs3a* in *kdm6a* ^+/−^ embryos. The results showed that enforced expression of *socs3a* could partially rescue the uncontrolled myeloid differentiation in these embryos (Figure 6F–I). Socs proteins can bind the Jak receptor to prevent Stat phosphorylation, thereby regulating the activation of the downstream Jak/Stat pathway, which plays multiple roles in hematopoietic system, including immune system homeostasis, differentiation and self‐renewal of HSPCs.^[^ ^42^ ^]^ Notably, GSEA identified upregulated expression of STAT3–targeted genes in *kdm6a* ^+/−^ HSPCs (Figure 6J). As determined by qPCR assay, expression levels of candidate Stat3 targets identified in GSEA, such as *ddx21*, *c1qbp* and *slc25a12*, were significantly increased in *kdm6a* ^+/−^ HSPCs. While ectopic expression of *kdm6a* in *kdm6a* ^+/−^ HSPCs attenuated these upregulated gene trends (Figure 6K). These data identify Kdm6a as an important regulator of Stat3 signaling activity in zebrafish. We further explored whether inhibition of JAK‐STAT pathway can reverse the phenotype of myeloid clone expansion. As expected, ruxolitinib and baricitinib, specific inhibitors of Jak, significantly reduced the number of myeloid cells in *kdm6a* ^+/−^ embryos (Figure 6L–O). Similarly, static, a specific inhibitor of Stat3 phosphorylation, also significantly reduced the number of myeloid cells in these embryos (Figure 6L–O). Taken together, our data indicate that Kdm6a haploinsufficiency promotes myeloid‐biased hematopoiesis through repressing *socs3a* expression and subsequently activating Jak‐Stat3 signaling in zebrafish.

### 2.6 A Conserved Role of KDM6A/SOCS3/p‐STAT3 Pathway in Regulating Myeloid Differentiation in Human HSPCs

**Figure 7:**
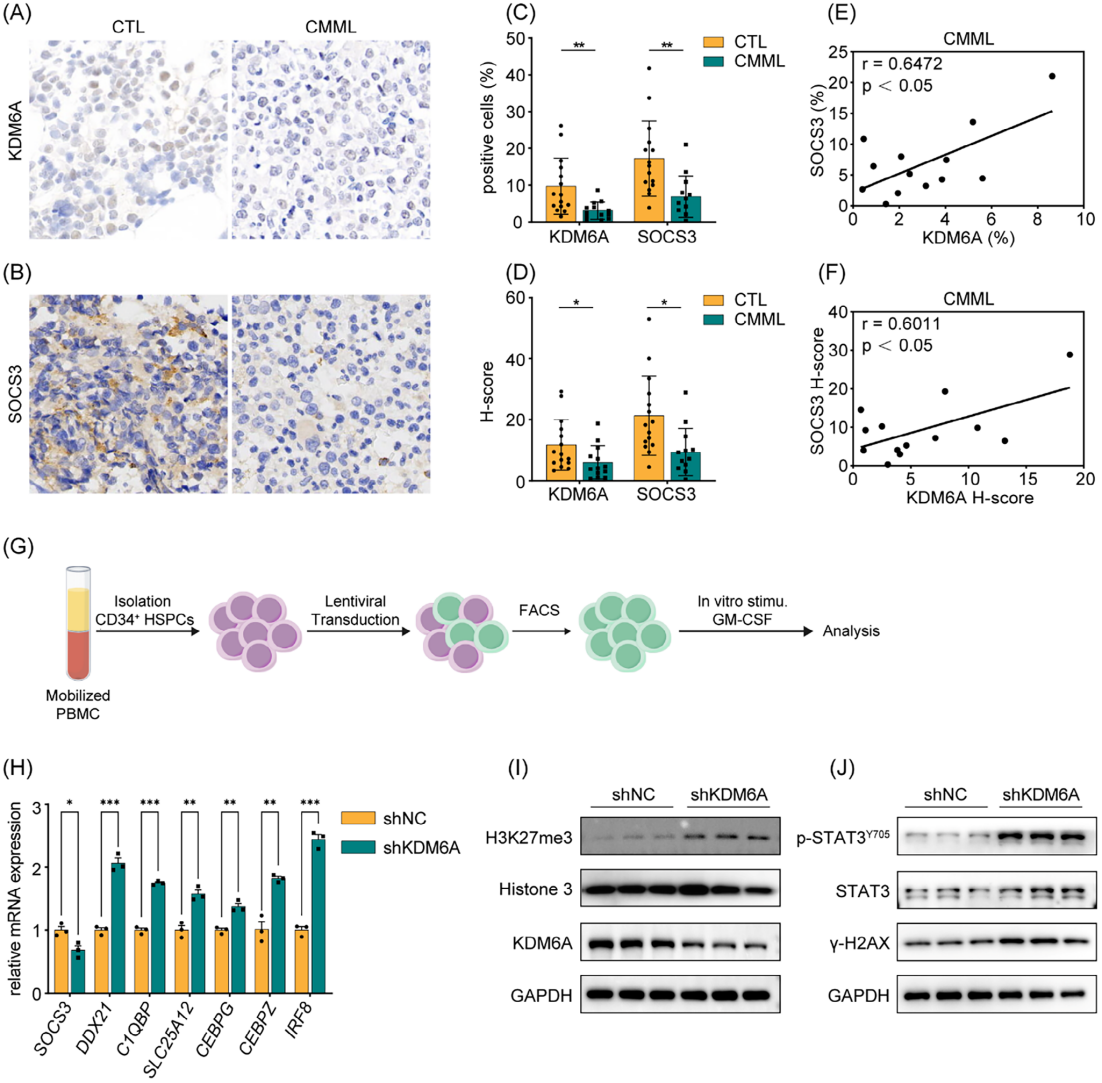
KDM6A/SOCS3/p‐STAT3 pathway is conserved in human HSPCs. A) Immunohistochemical staining of KDM6A on BM biopsies. B) Immunohistochemical staining of SOCS3 on BM biopsies. C) The percentage of KDM6A^+^ and SOCS3^+^cells in immunohistochemical staining. D) The H‐score of KDM6A^+^ and SOCS3^+^ cells immunohistochemical staining. E) Correlation analysis of the percentage of KDM6A^+^ (C, left) and SOCS3^+^ (C, right) cells in CMML specimens. F) Correlation analysis of the H‐score of KDM6A^+^ (D, left) and SOCS3^+^ (D, right) cells in CMML specimens. G) Schematic representation of human HSPC enrichment, lentiviral infection, and in vitro stimulation experiments conducted in this study. H) qPCR analysis of genes expression including *SOCS3*, *DDX21*, *C1QBP*, *SLC25A12*, *CEBPG*, *CEBPZ*, and *IRF8* in human HSPCs after GM‐CSF stimulation (*n* = 3, mean ± SD, Student's t test). I) Western Blot analysis of KDM6A, and H3K27me3 in human HSPCs after GM‐CSF stimulation. (J) Western Blot analysis of γH2AX, STAT3, and p‐STAT3^Y705^ in human HSPCs after GM‐CSF stimulation. *p <0.05; **p <0.01; ***p < 0.001.

Based on the findings that Kdm6a haploinsufficiency induces a monocytosis phenotype in zebrafish, which recapitulating key molecular features of CMML. We further investigated the pathogenic involvement of KDM6A/SOCS3 dysregulation in CMML. Immunohistochemical analysis of BM biopsies revealed significant downregulation of both KDM6A and SOCS3 in CMML patients compared with healthy controls (**Figure** **7**A–D). Furthermore, a positive correlation between SOCS3 and KDM6A expression was observed in CMML specimens (Figure 7E,F), consistent with the transcriptional suppression of *socs3a* observed in zebrafish with Kdm6a haploinsufficiency. Given the GM‐CSF hypersensitivity characteristic of CMML‐derived HSPCs and the GM‐CSF‐dependent monopoiesis process, we investigated HSPC homeostasis under hypercytokine conditions. HSPCs were enriched from healthy human mobilized peripheral blood mononuclear cells via immunomagnetic separation, followed by lentiviral shRNA‐mediated KDM6A knockdown (KD) (Figure 7G). GM‐CSF stimulation of KDM6A‐KD HSPCs resulted in *SOCS3* suppression, accompanied by the upregulation of STAT3 targets (*DDX21*, *C1QBP*, and *SLC25A12*), and master regulators of myeloid differentiation (*CEBPG*, *CEBPZ*, and *IRF8*) (Figure 7H). The evolutionary conserved KDM6A/SOCS3 regulatory axis indicates the preservation of mechanisms involved in myelopoiesis across species. Consistent with zebrafish, which demonstrate that KDM6A regulates the differentiation of HSPCs through its epigenetic activity, KDM6A‐KD HSPCs stimulated with GM‐CSF treatment exhibited increased expression of the H3K27me3 protein compared with control HSPCs. (Figure 7I). Importantly, elevated levels of γ‐H2AX and phosphorylated STAT3 (p‐STAT3^Y705^) were observed in KDM6A‐KD HSPCs (Figure 7J). These findings highlight the critical role of the KDM6A/p‐STAT3 pathway in myeloid differentiation across human and zebrafish models. Consistent with these findings, HSPCs derived from patients with CMML and subjected to KDM6A knockdown also showed increased p‐STAT3 levels and decreased SOCS3 protein expression (Figure S7A,B, Supporting Information). Furthermore, colony forming unit‐granulocyte macrophage (CFU‐GM) assays revealed that the JAK/STAT3 inhibitors significantly inhibited colony formation, and KDM6A‐KD cells exhibited increased sensitivity to JAK/STAT3 inhibitors (Figure S7C, Supporting Information). Collectively, these data establish the KDM6A/p‐STAT3 axis as a conserved and pivotal regulatory mechanism governing myeloid differentiation in both human and zebrafish systems, with potential therapeutic implications for CMML.

## 3 Discussion

Dysregulation of *KDM6A* gene expression has been identified in hematological malignancies, including AML, MDS, CMML, and even T‐ALL. Our study reveals three important aspects of KDM6A. First, we found that *kdm6a* ^−/−^ zebrafish exhibit reduced numbers of HSPCs and T cells, unveiling a critical role of Kdm6a as a positive regulator of HSPC emergence. Second, our results unbiasedly identified KDM6A deficiency as a distinct characteristic of CMML both in humans and zebrafish, highlighting the potential contribution of decreased expression of KDM6A to skewed myeloid differentiation. Third, we elucidated the molecular mechanisms by which KDM6A maintains HSPC generation and myeloid‐biased hematopoiesis.

Previous studies have shown that Kdm6a loss leads to enhanced self‐renewal capacity of HSCs, increasing the frequency and numbers of both long‐term HSCs and short‐term HSCs.^[^ ^43^ ^]^ In the present study, we revealed a novel role of Kdm6a in positively regulating HSPC generation as early as the embryonic stage through EHT process, suggesting that Kdm6a's regulatory functions are not confined to adult HSCs. It has been well established that inflammatory pathways play critical roles in EHT process. Several transmembrane pattern recognition receptor (PRR) pathways—including Toll‐like receptor (TLR) signaling, NOD‐like receptor (NLR) signaling, and RIG‐I‐like receptor (RLR) signaling—have been reported to regulate HSPC emergence.^[^ ^34^, ^44^, ^45^, ^46^ ^]^ Pro‐inflammatory cytokines, such as IL‐1, IL‐6, and TNFα, which are secreted upon activation of PRR pathways, have also been associated with HSPC development.^[^ ^32^, ^33^, ^47^ ^]^ Nevertheless, the mechanisms triggering these inflammatory pathways remain incompletely understood. Our results indicated that Kdm6a acts as a gatekeeper for the activation of inflammatory pathways during EHT, underscoring the interaction between epigenetic regulation and inflammatory signaling. Notably, ectopic expression of an enzymatically inactive Kdm6a variant failed to rescue the reduced numbers of HSPC in *kdm6a* ^−/−^ zebrafish, suggesting that Kdm6a stimulates HSPC formation from ECs by activating inflammatory pathways in a demethylase‐dependent manner. Additionally, CLR signaling, a member of PRR pathways, participates in the recognition and initiation of adaptive immune responses by producing various cytokines, including IL‐1β, IL‐12, IL‐6, IL‐23, interferon‐β (IFN‐β), and TNF, as well as the chemokines CXCL1 and CXCL2.^[^ ^48^ ^]^ Consistent with previous evidence indicating that loss of KDM6A reduces cytokine production,^[^ ^49^ ^]^ we observed decreased expression levels of *il‐6*, *il‐1b*, and *tnfa*. A notable downregulation of *syk*, a critical signaling molecule in the CLR signaling,^[^ ^50^, ^51^ ^]^ was also observed in *kdm6a* ^−/−^ ECs. Furthermore, the induced expression of *syk* partially rescued these HSPC defects in *kdm6a* ^−/−^ zebrafish. Taken together, our study suggested that Kdm6a‐depentent activation of the CLR pathway plays critical roles at the EHT stage, providing new insights into the molecular mechanisms governing HSPC generation. On the other hand, our RNA‐seq results revealed the inhibition of other PRR pathways in Kdm6a‐deficient ECs, suggesting that Kdm6a regulates HSPC generation through multiple PRR pathways.

Our studies further established an endogenous Kdm6a‐haploinsufficient model that simulates characteristics of *KDM6A* mutations identified in female patients with hematologic malignancies. Owing to the dysregulation of hematopoiesis, the incidence of hematologic malignancies increases during aging. Whether interventions to prevent HSC aging or rejuvenate the functionality of aged HSCs can effectively prevent leukemia development remains an open question. Previous studies reported that KDM6A plays a crucial role in preventing aging progression in murine hematopoietic system, via both demethylase‐dependent and ‐independent mechanisms.^[^ ^52^ ^]^ Our study added evidence to the role of KDM6A in aging, showing that haploinsufficiency of Kdm6a induces phenotypes of hematopoietic aging characterized by myeloid skewing, observed as early as the embryonic stage in zebrafish models. Furthermore, some adult zebrafish with Kdm6a‐haploinsufficient progressed to CMML‐like phenotypes, consistent with reduced levels of KDM6A protein observed in bone marrow cells from our CMML patients. Additionally, it has shown that haploinsufficiency of KDM6A results in diminished functionality of NK cells.^[^ ^14^, ^23^ ^]^ Loss of KDM6A inhibits CD8^+^ T‐cell‐dependent antitumor immune responses.^[^ ^49^ ^]^ Taken together, both published data and our data illustrate that KDM6A has a profound effect on normal hematopoietic differentiation, especially lineage balance between myeloid and lymphoid differentiation.

JAK/STAT pathway regulates various cellular functions, including proliferation, apoptosis, differentiation, and aging, thereby contributing to the maintenance of hematologic homeostasis.^[^ ^53^, ^54^, ^55^ ^]^ Although inhibition of the JAK/STAT pathway has been explored as a therapeutic approach for CMML, existing studies mostly focus on the constitutive activation of the pathway due to acquired mutations, such as JAK2^V617F^. To date, there have been no reports linking *KDM6A* mutations to the promotion of CMML via the JAK/STAT3 pathway. Our study identified *SOCS3* as a target gene of KDM6A and clarifies the role of the JAK/STAT3 pathway as a downstream effector in KDM6A‐mediated myeloid differentiation, specifically in an in vivo zebrafish model. This finding addresses a critical gap in understanding the mechanisms by which *KDM6A* mutations or deficiencies contribute to CMML pathogenesis. Ruxolitinib, a first‐generation JAK1/2 inhibitor, has demonstrated limited efficacy in CMML, with current clinical research indicating an overall response rate of merely 35%.^[^ ^56^ ^]^ Our study pioneers the therapeutic potential of Baricitinib, a second‐generation selective JAK inhibitor, for CMML. Existing research on Baricitinib in the context of rheumatoid arthritis indicates favorable safety and efficacy profiles.^[^ ^57^, ^58^ ^]^ Consequently, Baricitinib may, in the future, supersede Ruxolitinib as a more tolerable JAK inhibitor for CMML treatment. Additionally, previous research has focused more on the role of STAT5 phosphorylation in CMML,^[^ ^59^ ^]^ with a notable absence of investigations into the application of STAT3 inhibitors, such as STATTIC. Our research not only validates the efficacy of STAT3 inhibitors as monotherapy but may also propose a “dual blockade” (JAK inhibitor + STAT3 inhibitor) strategy for the JAK/STAT3 pathway, enhancing anti‐leukemic effects through synergistic action upstream and downstream, and reducing the risk of drug resistance.

In summary, our findings demonstrate that KMD6A bridges inflammation pathways and HSPC generation by regulating H3K27me3 levels in a demethylase‐dependent manner. A decreased expression of KMD6A enhances the myeloid differentiation preference and alters the chromatin accessibility of genes related to aging and cellular homeostasis in HSPCs. KDM6A deficiency promotes myeloid‐biased hematopoiesis by activation of SOCS3/JAK/STAT3 pathway. Further studies are necessary to clarify how KDM6A recognizes inflammation‐related and aging‐related genes, and whether the inhibition of H3K27me3 may prevent leukemic transformation or offer potential therapeutic benefits for CMML.

## 4 Experimental Section

### Zebrafish Husbandry and Transgenic Strains

Zebrafish maintenance and staging were performed as described previously.^[^ ^60^ ^]^ The following transgenic lines were used in this study: wild‐type (WT) AB strain, Tg(*flia*:eGFP), Tg(*runx1*:eGFP), Tg(*kdrl*:mCherry), Tg(*cmyb*:eGFP), and Tg(*cd41*:eGFP). The study was approved by the Ethics Committee of Zhejiang University (No. ZJU20250097) and the methods were carried out in accordance with the approved guidelines.

### Generation of *kdm6a*‐Mutant Lines and Validation

To generate targeted disruptions in the *kdm6a* genomic locus, the CRISPR/Cas9 system was employed. Briefly, recombinant Cas9 protein (New England Biolabs, M0646T) and 50pg gRNA were injected into single‐cell WT embryos. The injected F0 founders were raised to maturity and identified with inheritable mutations. F1 progeny from F0 outcrosses was identified through genotyping and sequencing. Identified F1 and their progeny were used for experiments. *kdm6a* ^+/+^ and *kdm6a* ^−/−^ zebrafish were generated and genotyped from heterozygous intercrosse.

### shRNA, Morpholino Oligonucleotide, mRNA Synthesis and Plasmid Construction

Lentiviruses expressing KDM6A shRNA (5′‐TTTATCAATAGACTGCCTGTA‐3′)^[^ ^26^ ^]^ was generated by cloning into the pLKO.1‐ZsGreen vector. The antisense atgMO against zebrafish *kdm6a* (5′‐CCACCGACACTCCGCACGATTTCAT‐3′)^[^ ^31^ ^]^ was purchased from Gene Tools. Capped mRNAs were synthesized from linearized plasmids using the mMessage mMachine SP6 kit (Invitrogen, AM1340) and diluted to 50–150 ng µl^−1^ for microinjection. The full‐length coding DNA sequence of *syk* was cloned into pDestTol2pA2 using the *kdrl* promoter and a mCherry reporter using HiFi DNA Assembly Master Mix (New England Biolabs, E2621S). MO, mRNA and constructs with *tol2* mRNA were injected into zebrafish embryos at single‐cell stage.

### Whole‐Mount In Situ Hybridization and Flow Cytometry

Whole mount in situ hybridization was performed as described previously. Digoxigenin‐labeled RNA probes were transcribed with T7 (Promega, P2075), T3 (Promega, P2083), or SP6 polymerase (Invitrogen, AM1340). Probes were detected using alkaline phosphatase (AP)‐coupled anti‐digoxigenin Fab fragment antibody (Roche, 11 093 274 910) with BCIP/NBT staining (Solarbio, PR1100). The stained embryos were then photographed using a stereomicroscope (Nikon) equipped with a digital camera.

Transgenic embryos were dissociated into single cells using Dispase II (Yuanye, S25046). Single‐cell suspension was obtained by centrifugation at 400 g for 5 min, washing twice with PBS, and passing through a 40 µm nylon mesh filter. Then 1 µl 7AAD (Biolegend, 420404) was added to the samples immediately before the flow cytometric analysis. The number of fluorescence‐labeled cells was then determined on a Beckman CytoFlex S flow cytometer, and data were analyzed using FlowJo software.

### Gene Expression Analysis using qPCR

For zebrafish, total RNA was extracted from the dissected trunk regions of embryos using TRIzol reagent (Thermo Fisher Scientific, 15 596 026) or from sorted cells using RNeasy Micro Kit (Qiagen, 74 004). For human cells, total RNA was extracted from the cells using TRIzol reagent (Thermo Fisher Scientific, 15 596 026). The mRNA was reverse transcribed using HiScript II Reverse Transcriptase (Vazyme, R223‐01). qPCR was with Champagne Taq DNA Polymerase (Vazyme, Q711) and fold‐changes determined by ΔΔCt method.

### Wright‐Giemsa Staining

Kidney marrow cells were resuspended in ice‐cold phosphate‐buffered saline (PBS) containing 5% fetal bovine serum. A total of 5×10^5^ resuspended cells were spun onto slides using cytospins, 400 rpm, 3 min. The slides were then stained with Wright‐Giemsa Stain Kit (Solarbio, G1021), washed with water, air‐dried, and then examined under a microscope. Images were captured using Nikon Ni microscope (Nikon).

### RNA Sequencing (RNA‐seq), Smart‐Seq 2 and Bioinformatics

At 28 h postfertilization (hpf), endothelial cells (*flia*:eGFP) from trunk region in *kdm6a* morphants and their WT controls were sorted using flow cytometry (Beckman Coulter, MoFlo XDP). Total RNA was extracted from these sorted eGFP^+^ cells by using TRIzol Reagent (Thermo Fisher Scientific, 15 596 018) following the manufacturer's instructions. Sequencing libraries were prepared by SuperScript^TM^ II Reverse Transcriptase (Invitrogen, 1 896 649). Sequencing was by an illumina Novaseq^TM^ 6000 platform (LC Bio Technology CO.,Ltd. Hangzhou, China) to obtain paired‐end sequencing (PE150) reads. At 3 days postfertilization (dpf), equal amount (200 cells) of hematopoietic stem and progenitor cells (*cd41*:eGFP) from caudal hematopoietic tissue region in *kdm6a* ^+/−^ and their WT controls were sorted using flow cytometry (Beckman Coulter, MoFlo XDP) and collected into 4 µl of lysis buffer. RNA‐seq library construction for low input cells was carried out as previously described.^[^ ^61^ ^]^ Sequencing was by an DNBSEQ^TM^ platform (BGI Technology CO.,Ltd. Shenzhen, China) to obtain paired‐end sequencing (PE100) reads.

After removing the low‐quality bases and undetermined bases by Cutadapt (version: cutadapt‐1.9), We aligned reads of all samples to the *Danio reio* reference genome (GRCz11) using HISAT2 (version: hisat2‐2.2.1) package. The mapped reads of each sample were assembled using StringTie (version: stringtie‐2.1.6) with default parameters. Then, all transcriptomes from all samples were merged to reconstruct a comprehensive transcriptome by using gffcompare software (version: gffcompare‐0.9.8.). After the final transcriptome was generated, StringTie and ballgown were used to estimate the expression levels of all transcripts and perform expression level for mRNAs by calculating FPKM. Genes differential expression analysis was performed by DESeq2 software between two different groups. The genes with the parameter of p‐val<0.05 and absolute fold change ≥ 1.5 were considered differentially expressed genes. Differentially expressed genes were then subjected to enrichment analysis of GO functions and KEGG pathways. We performed gene set enrichment analysis using software GSEA (v4.1.0) meeting this condition with |NES|>1, NOM p‐val<0.05, FDR q‐val<0.25 considered to be different in two groups.

### Transposase‐Accessible Chromatin Sequencing (ATAC‐seq)

Using TruePrep^TM^ DNA library Prep Kit V2 for Illumina (Vazyme, TD501), ≈20 000 cells per sample were used for ATAC‐seq according to the manufacturer's instructions. The sorted cells were washed once in 1×PBST, and then lysed in 50 µl pre‐cooling lysis buffer for 5 min on ice. The transposition reaction system with 10 µl 5×TTBL, 5 µl TTE Mix, and 33 µl H2O were added to the sample and incubated at 37 °C for 30 min. After that, the DNA was purified with chloroform‐phenol and amplified using TruePrep^TM^ DNA Index Kit V2 for Illumina (Vazyme, TD202). The DNA library was purified using VAHTS^TM^ DNA Clean Beads (Vazyme, N411), and then sequenced under Illumina NovaSeq 6000 platform with paired‐end (PE150) reads.

Adaptor sequences were trimmed with Trimmomatic (v0.36) and the Bowtie2 was used for aligning ATAC‐seq reads to the zebrafish genome version GRCz11. Samtools (version 0.1.19) were used for data filtering and file format conversion. Duplicate reads, and chrM were removed before peak calling. Gene annotation (100 kb upstream and 50 kb downstream of the TSS) and genomic distribution of accessible regions identified by MACS2 was performed with BEDTools and ‐closetBed and ‐intersectBed subcommands respectively.

### Chromatin Immunoprecipitation (ChIP) Assay

ChIP assay was performed with SimpleChIP Plus Enzymatic Chromatin IP Kit (Cell Signaling Technology, 9005). Briefly, zebrafish embryos were harvested and crosslinked with 1.5% formaldehyde for 20 min at room temperature. After sonication, the soluble chromatins were incubated with the following antibodies separately: H3K27me3 (Cell Signaling Technology, 9733) or control IgG (Cell Signaling Technology, 2729). The immunoprecipitated complex was washed, and DNA was extracted and purified. ChIP DNA was analyzed by qPCR, and the data were normalized to input DNA. The primers used for ChIP‐qPCR were listed in Table S1 (Supporting Information).

### Inhibitor Treatment

Embryos were soaked in egg water and treated with 20 µM Ruxolitinib (TargetMol, T1829), Baricitinib (TargetMol, T2485), or Stattic (TargetMol, T6308) from 48 to 72 hpf.

### Immunohistochemical Staining

Tissue sections were deparaffinized and rehydrated using a series of xylene and ethanol washes. Antigen retrieval was performed by heating the sections in citrate buffer (pH 6.0) at 95 °C for 20 min. The sections were then allowed to cool to room temperature and subsequently washed with phosphate‐buffered saline (PBS). To block endogenous peroxidase activity, sections were incubated with 3% hydrogen peroxide in methanol for 10 min. Non‐specific binding sites were blocked by incubating the sections with 5% bovine serum albumin (BSA) in PBS for 30 min at room temperature. KDM6A (Cell Signaling Technology, 33 510) and SOCS3 (Abcam, ab280884) antibody were applied to the sections and incubated overnight at 4 °C in a humidified chamber. After washing with PBS, secondary antibodies conjugated to horseradish peroxidase (HRP) were applied and incubated for 1 h at room temperature. The sections were then washed again with PBS. The HRP enzyme activity was visualized using 3,3′‐diaminobenzidine (DAB) as the chromogen, according to the manufacturer's instructions. Sections were counterstained with hematoxylin, dehydrated through graded alcohols and xylene, and mounted with a coverslip.

### Isolation and Stimulation of CD34^+^ Hematopoietic Stem and Progenitor Cells

Human mobilized peripheral blood samples from healthy donor, and bone marrow samples from CMML patients were collected at the Sir Run Run Shaw Hospital upon availability. Written informed consent was obtained from the patient, and the study were approved by the Ethics Committee of Sir Run Run Shaw Hospital of Zhejiang University School of Medicine (No. 2023‐0345). Mononuclear cells were enriched by using a Ficoll gradient, and CD34^+^ cells were purified by using Human CD34 Positive Selection Kit II (Stemcell Technologies, 17 856). Purified HSPCs were cultured for less than 48 h in StemSpan™ SFEM II (Stemcell Technologies, 0 9605) supplemented with Expansion Supplement (Stemcell Technologies, 0 2691). Then the cells were lentiviral transduced with the previously described shKDM6A or negative control plasmids according to standard protocols. After sorting ZsGreen‐positive cells using flow cytometry, these cells were stimulated with 10 ng mL^−1^ GM‐CSF (Pepro Tech, 300–03) for 15 min, and subsequently collected for qPCR and WB analyses.

### Western Blot Analysis

The cells were collected and homogenized in RIPA. The blots were performed using standard methodology with the following antibodies: GAPDH (Cell Signaling Technology, 2118), KDM6A (Cell Signaling Technology, 33 510), Histone H3 (Cell Signaling Technology, 4499), H3K27me3 (Cell Signaling Technology, 9733), SOCS3 (Abcam, ab280884), STAT3 (Cell Signaling Technology, 12 640), phospho‐STAT3 (Tyr705) (Cell Signaling Technology, 9145), and γH2AX (GeneTex, GTX127342).

### Colony Forming Assays

For CFU assays, 1000 CD34^+^ treated or not with the inhibitors were seeded in duplicate in 35×10‐mm dishes containing 1.5 mL of methylcellulose (H4230, StemCell), supplemented with 1% P/S, FLT3‐L (25 ng µL^−1^), THPO (25 ng µL^−1^), SCF (60 ng µL^−1^), IL3 (10 ng mL^−1^), GM‐CSF (10 ng mL^−1^).Colonies were counted after 14 days of culture.

### Statistical Analysis

All statistical analysis was performed for at least 3 independent biological repeats. GraphPad Prism 9 was used to analyze the data. Data are mean ± standard deviation (SD). P values calculated by 2‐tailed, unpaired Student t test was used to indicate the significance if not clarified in figure legends. *P < 0.05; **P < 0.01; ***P < 0.001.

## Conflict of Interest

The authors declare no conflict of interest.

## Author Contributions

H.C., S.W., and R.D. contributed equally to this work. H.W.X., and H.Q.C. designed the research study and analyzed the data; H.Q.C., S.F.W., R.Y. D., P.H.Y., and T.Y.L. performed the experiments and collected the data; L.N.H., M.W.W., Z.J. Q., H.Y.Z., X.Y.Y., and L.M.W. provided suggestions on experimental design and data presentation; H.Q.C. wrote the paper and H.W.X. revised the paper. All authors read and approved the final manuscript.

## Supporting information



Supporting Information

## Data Availability

The sequence data reported in this paper have been deposited in the Genome Sequence Archive (Genomics, Proteomics & Bioinformatics 2021) in National Genomics Data Center (Nucleic Acids Res 2022), China National Center for Bioinformation / Beijing Institute of Genomics, Chinese Academy of Sciences (GSA: CRA019121) that are publicly accessible at https://ngdc.cncb.ac.cn/gsa.
