# Supplementary material for: KDM6A Deficiency Induces Myeloid Bias and Promotes CMML‐Like Disease Through JAK/STAT3 Activation by Repressing SOCS3
Source: Adv Sci (Weinh). 2025 May 14;12(21):2413091. doi: 10.1002/advs.202413091 (PMC12140365; doi:10.1002/advs.202413091)
Supplement: Supplementary file 1 — Supporting Information [file ADVS-12-2413091-s001.pdf]

ADVANCED SCIENCE
Open Access

Supporting Information

for *Adv. Sci.*, DOI 10.1002/advs.202413091

KDM6A Deficiency Induces Myeloid Bias and Promotes CMML-Like Disease Through JAK/STAT3 Activation by Repressing SOCS3

*Huiqiao Chen, Shufen Wang, Ruoyu Dong, Pinhui Yu, Tianyu Li, Liangning Hu, Mowang Wang, Zijun Qian, Hongyu Zhou, Xiaoyan Yue, Limengmeng Wang and Haowen Xiao**

KDM6A Deficiency Induces Myeloid Bias and Promotes CMML-like Disease through JAK/STAT3 Activation by Repressing SOCS3

Huiqiao Chen[1], *Shufen Wang*[1], *Ruoyu Dong*[1], *Pinghui Yu*[1], *Tianyu Li*[1], *Liangning Hu*[1], *Mowang Wang*[2], *Zijun Qian*[1], *Hongyu Zhou*[1], *Xiaoyan Yue*[1], *Limengmeng Wang*[2, 3], *Haowen Xiao*[1, 3]*

[1]Department of Hematology and Cell Therapy, Sir Run Run Shaw Hospital, Zhejiang University School of Medicine, Hangzhou, Zhejiang province, P.R. China.

[2]Bone Marrow Transplantation Center, The First Affiliated Hospital, Zhejiang University School of Medicine, Hangzhou, Zhejiang province, P.R. China.

[3]Institute of Hematology, Zhejiang University, Hangzhou, Zhejiang province, P.R. China.

Statement of equal author contributions: Huiqiao Chen, Shufen Wang, and Ruoyu Dong contributed equally to the study.

*Correspondence to:

Haowen Xiao, Ph.D., M.D., Department of Hematology and Cell Therapy, Sir Run Run Shaw Hospital, Zhejiang University School of Medicine, No. 3 Qingchun East Rd., Hangzhou, 310016, Zhejiang province, P.R. China.

Tel: +86 571 86006936; Fax: +86 571 86006936.

E-mail: haowenxiaoxiao@zju.edu.cn

Supplemental Figure Legends

Figure S1. Multiple alignments of KDM6A conserved domains from human and zebrafish.

(**A**) Conserved TPRs domain from human and zebrafish.

(**B**) Conserved JmjC domain from human and zebrafish.

Figure S2. Genomic editing using CRISPR/Cas9 to generate *kdm6a* mutant zebrafish.

(**A**) Site-specific targeting for CRISPR/Cas9 cleavage within the zebrafish *kdm6a* gene. PAM protospacer adjacent motif.

(**B-C**) Alignment of nucleotide sequences from wild-type and mutant *kdm6a* alleles in *kdm6a* $^{e10\ (\triangle 10)}$ zebrafish line. Dashes in DNA sequences are the nucleotides deleted during repair of CRISPR/Cas9-induced double-strand breaks. CRISPR/cas9-induced *kdm6a* frameshift mutations predicted to lead to truncated protein destroying the TPRs domain and lacking the JmjC domain.

(**C**) Alignment of nucleotide sequences from wild-type and mutant *kdm6a* alleles in *kdm6a* $^{e23\ (\triangle 1)}$ zebrafish line. Dashes in DNA sequences are the nucleotides deleted during repair of CRISPR/Cas9-induced double-strand breaks. CRISPR/cas9-induced *kdm6a* frameshift mutations predicted to lead to truncated protein destroying the JmjC domain.

(**D and F**) The survival of *kdm6a* $^{e10\ (\triangle 10/\triangle 10)}$ zebrafish and their siblings at different developmental stages.

(**E and G**) The survival of *kdm6a* $^{23\ (\triangle 1/\triangle 1)}$ zebrafish and their siblings at different developmental stages.

Figure S3. *kdm6a* is required for HSPC emergence in zebrafish.

(**A**) Western Blot analysis of KDM6A using lysates from control and *kdm6a* MO embryos at 28 hpf.

(**B**) WISH for *runx1* in embryos at 28 hpf. The ratio of embryos with the representative expression pattern is indicated at the right bottom.

(**C**) WISH for *runx1* in embryos at 30 hpf. The ratio of embryos with the representative expression pattern is indicated at the right bottom.

(**D**) WISH for *runx1* in embryos at 36 hpf. The ratio of embryos with the representative expression pattern is indicated at the right bottom.

(**E**) WISH for *cmyb* in embryos at 3 dpf. The ratio of embryos with the representative expression pattern is indicated at the right bottom.

(**F**) Representative images of *rag2*$^{+}$ cells in Tg (*rag2*:eGFP) embryos in the thymus of control embryos and *kdm6a* morphants at 4 dpf.

(**G**) Representative images of *coro1a*$^{+}$ cells in Tg (*coro1a*:eGFP) embryos in the thymus of control embryos and *kdm6a* morphants at 4 dpf.

(**H**) qPCR analysis of *runx1* and *cmyb* expression in control embryos and *kdm6a* morphants at different developmental stages (n = 3, mean ± SD, Student's t test).

Figure S4. Kdm6a-deficient embryos displayed normal vasculogenesis.

(**A**) WISH for *kdrl* in embryos at 28 hpf. The ratio of embryos with the representative expression pattern is indicated at the right bottom.

(**B**) WISH for *dlc* in embryos at 28 hpf. The ratio of embryos with the representative expression pattern is indicated at the right bottom.

(**C**) WISH for *tbx20* in embryos at 28 hpf. The ratio of embryos with the representative expression pattern is indicated at the right bottom.

(**D**) Confocal imaging showing the endothelial marker Tg (*fli1a*:eGFP) at 28 hpf. The ratio of embryos with the representative expression pattern is indicated at the right bottom.

Figure S5. *syk* is required for HSPC emergence in zebrafish.

(**A**) Genomic editing using CRISPR/Cas9 to generate *syk* CRISPRi zebrafish.

(**B**) WISH for *runx1* in embryos at 28 hpf. The ratio of embryos with the representative expression pattern is indicated at the right bottom.

(**C**) WISH for *cmyb* in embryos at 3 dpf. The ratio of embryos with the representative expression pattern is indicated at the right bottom.

Figure S6. Deficiency of Socs3a has skewed myelopoiesis in embryonic zebrafish.

(**A**) Genomic editing using CRISPR/Cas9 to generate *socs3a* CRISPRi zebrafish.

(**B**) WISH for *mfap4* in embryos at 3 hpf. The ratio of embryos with the representative expression pattern is indicated at the right bottom.

(**C**) WISH for *hbae1* in embryos at 3 dpf. The ratio of embryos with the representative expression pattern is indicated at the right bottom.

(**D**) WISH for *mpx* in embryos at 3 dpf. The ratio of embryos with the representative expression pattern is indicated at the right bottom.

(**E**) WISH for *lyz* in embryos at 3 dpf. The ratio of embryos with the representative expression pattern is indicated at the right bottom.

Figure S7. A conserved role of KDM6A/SOCS3/p-STAT3 pathway in HSPCs derived from patients with CMML.

(**A**) Schematic representation of HSPC enrichment, lentiviral infection, and in vitro stimulation experiments conducted in this study.

(**B**) Western Blot analysis of KDM6A, SOCS3, STAT3, and p-STAT3^{Y705} in CMML HSPCs after GM-CSF stumilation.

(**C**) CFU-GM analysis of KDM6A-KD HSPCs and control HSPCs in the presence of JAK/STAT3 inhibitors.

Figure S1

(A)

TPRs

```
     human: SDFFCQLGHFNLLLEDYPKALSAYQRYYSLQSDYWKNAAFLYGLGLVYFH
 zebrafish: PEVFCQLGHFNLLLEDYPKALSAYQRYYSLQSDYWKNAAFLYGLGLVYFH
 Alignment:    FCQLGHFNLLLEDYPKALSAYQRYYSLQSDYWKNAAFLYGLGLVYFH

     human: YNAFQWAIKAFQEVLYVDPSFCRAKEIHLRLGLMFKVNTDYESSLKHFQL
 zebrafish: YNAFRWAIKAFQEVLYIDPCFSRAKEIHLRLGLMFKVNTDYESSLKHFQL
 Alignment: YNAF WAIKAFQEVLY6DP F RAKEIHLRLGLMFKVNTDYESSLKHFQL

     human: ALVDCNPCTLSNAEIQFHIAHLYETQRKYHSAKEAYEQLLQTENLSAQVK
 zebrafish: ALIDSTPCTLSKAEIQFHIAHVYEIQKKYRIAKEAYESLLQTENLPAQVK
 Alignment: AL6D  PCTLS AEIQFHIAH6YE Q4KY  AKEAYE LLQTENL AQVK

     human: ATVLQQLGWMHHTVDLLGDKATKESYAIQYLQKSLEADPNSGQSWYFLGR
 zebrafish: ATTLQQLGWMHHTVEQLGDKANKNSYAIQCLQKSLEADPNSGQSWYFLGR
 Alignment: AT LQQLGWMHHTV  LGDKA K SYAIQ LQKSLEADPNSGQSWYFLGR

     human: CYSSIGKVQDAFISYRQSIDKSEASADTWCSIGVLYQQQNQPMDALQAYI
 zebrafish: CYSSIGKVQDAFISYRQSIDKSEASADTWCSIGVLYQQQNQPMDALQAYI
 Alignment: CYSSIGKVQDAFISYRQSIDKSEASADTWCSIGVLYQQQNQPMDALQAYI

     human: CAVQLDHGHAAAWMDLGTLYESCNQPQDAIKCYLNA
 zebrafish: CAVQLDHSHAAAWMDLGTLYESCNQPQDAIKCYINA
 Alignment: CAVQLDH HAAAWMDLGTLYESCNQPQDAIKCY6NA
```

(B)

JmjC

```
     human: QLHELTKLPAFVRVVSAGNLLSHVGHTILGMNTVQLYMKVPGSRTPGHQE
 zebrafish: QLAELSKLPAFARVVSAGNLLSHVGHTILGMNTVQLYMKVPGSRTPGHQE
 Alignment: QL EL3KLPAF RVVSAGNLLSHVGHTILGMNTVQLYMKVPGSRTPGHQE

     human: NNNFCSVNINIGPGDCEWFVVPEGYWGVLNDFCEKNNLNFLMGSWWPNLE
 zebrafish: NNNFCSVNINIGPGDCEWFAVPEPYWGVMNDFCEKNNINYLMGSWWPNLE
 Alignment: NNNFCSVNINIGPGDCEWF VPE YWGV6NDFCEKNN6N5LMGSWWPNLE

     human: DLYEANVPVYRFIQRPGDLVWINAGTVHWVQAIGWCNNIAWNV
 zebrafish: DLYEANVPVYRFIQRPGDLVWLNTGTVHWVQAIGWCNNIAWNV
 Alignment: DLYEANVPVYRFIQRPGDLVW6N GTVHWVQAIGWCNNIAWNV
```

Figure S2

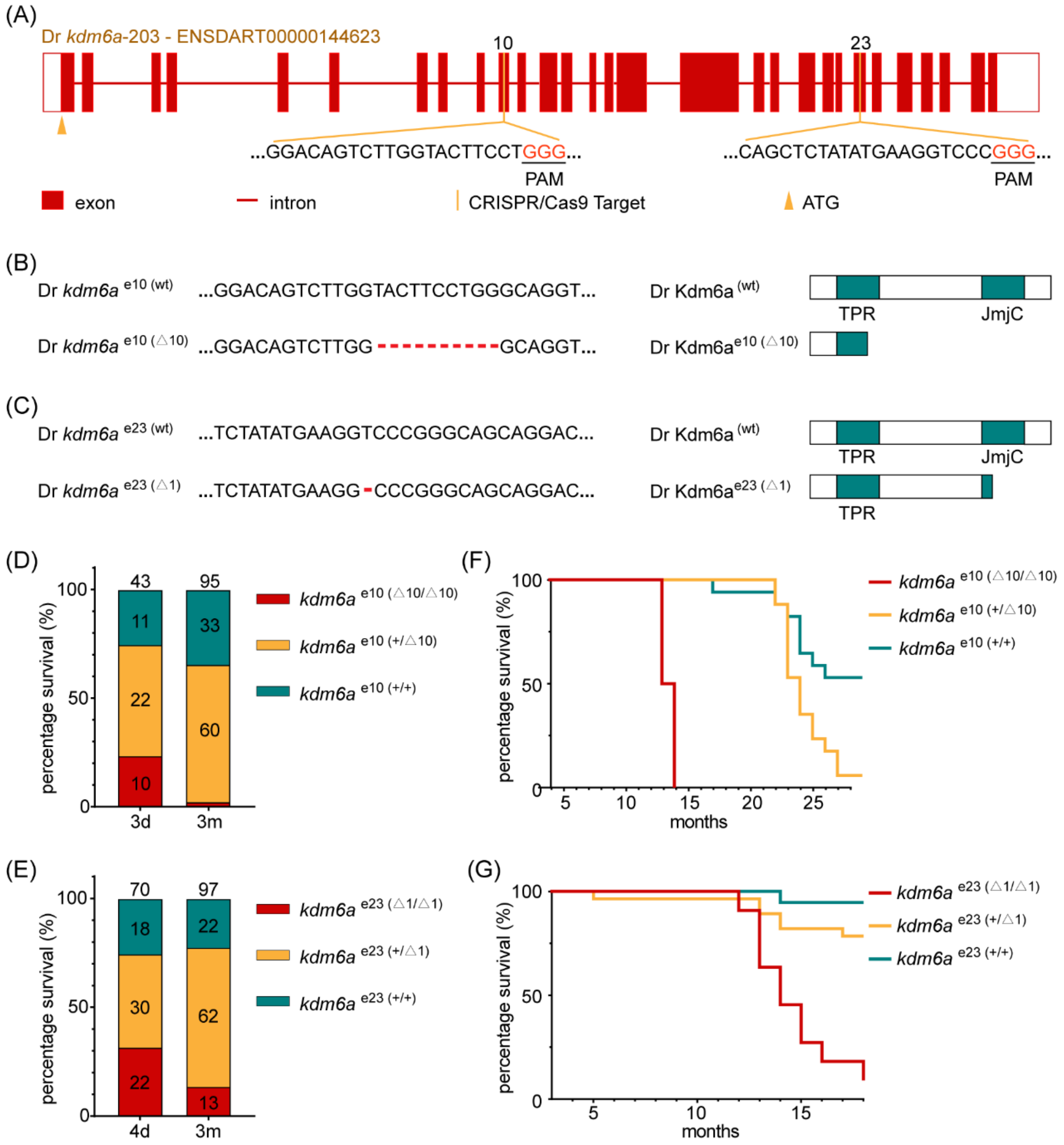

(A)
Dr kdm6a-203 - ENSDART00000144623
10
23
...GGACAGTCTTGGTACTTCCTGGG...
PAM
...CAGCTCTATATGAAGGTCCCGGG...
PAM
exon
intron
CRISPR/Cas9 Target
ATG
(B)
Dr kdm6a e10 (wt)
...GGACAGTCTTGGTACTTCCTGGGCAGGT...
Dr Kdm6a (wt)
TPR
JmjC
Dr kdm6a e10 (△10)
...GGACAGTCTTGG----------GCAGGT...
Dr Kdm6a e10 (△10)
(C)
Dr kdm6a e23 (wt)
...TCTATATGAAGGTCCCGGGCAGCAGGAC...
Dr Kdm6a (wt)
TPR
JmjC
Dr kdm6a e23 (△1)
...TCTATATGAAGG-CCCGGGCAGCAGGAC...
Dr Kdm6a e23 (△1)
TPR
(D)
percentage survival (%)
100
50
0
43
95
11
33
22
60
10
3d
3m
kdm6a e10 (△10/△10)
kdm6a e10 (+/△10)
kdm6a e10 (+/+)
(F)
percentage survival (%)
100
50
0
5
10
15
20
25
months
kdm6a e10 (△10/△10)
kdm6a e10 (+/△10)
kdm6a e10 (+/+)
(E)
percentage survival (%)
100
50
0
70
97
18
22
30
62
22
13
4d
3m
kdm6a e23 (△1/△1)
kdm6a e23 (+/△1)
kdm6a e23 (+/+)
(G)
percentage survival (%)
100
50
0
5
10
15
months
kdm6a e23 (△1/△1)
kdm6a e23 (+/△1)
kdm6a e23 (+/+)

Figure S3

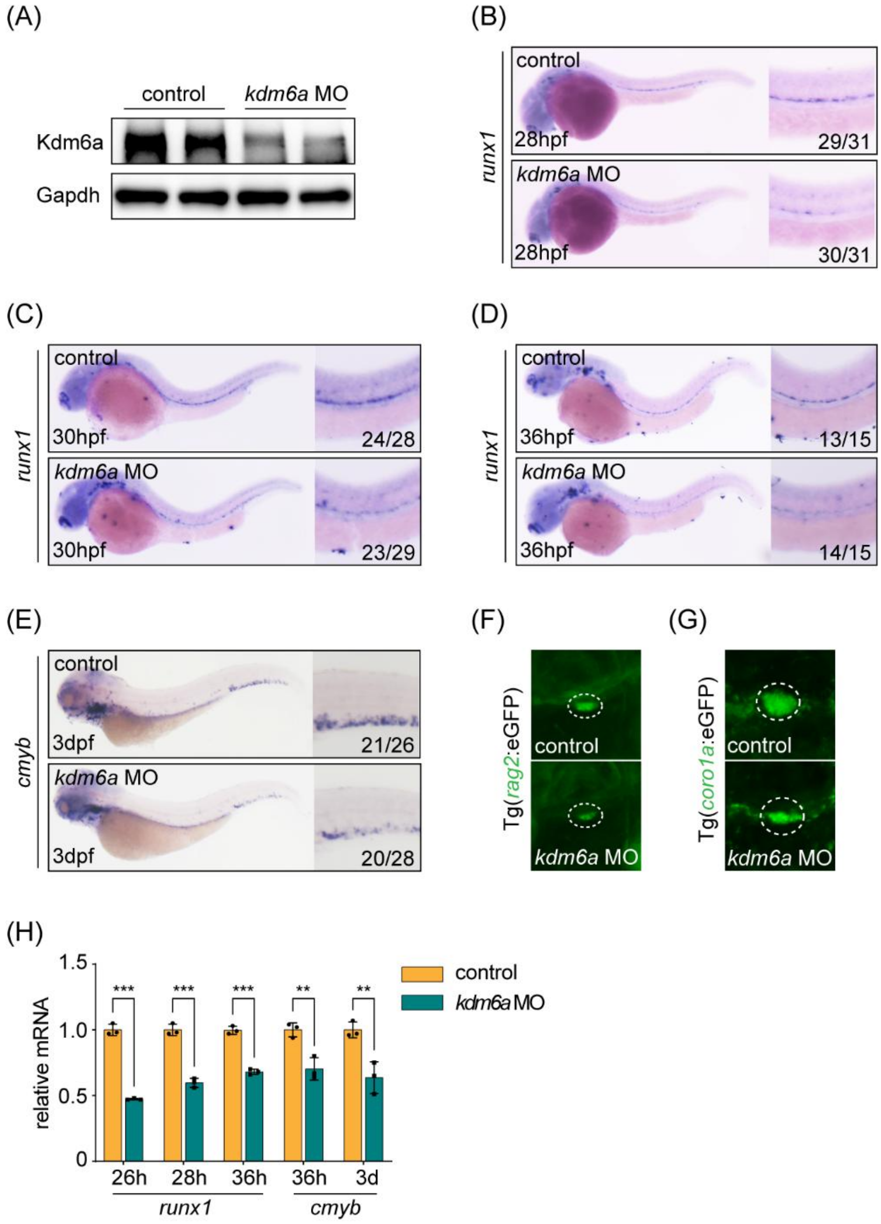

(A)
control
kdm6a MO
Kdm6a
Gapdh
(B)
runx1
control
28hpf
29/31
kdm6a MO
28hpf
30/31
(C)
runx1
control
30hpf
24/28
kdm6a MO
30hpf
23/29
(D)
runx1
control
36hpf
13/15
kdm6a MO
36hpf
14/15
(E)
cmyb
control
3dpf
21/26
kdm6a MO
3dpf
20/28
(F)
Tg(rag2:eGFP)
control
kdm6a MO
(G)
Tg(coro1a:eGFP)
control
kdm6a MO
(H)
relative mRNA
1.5
1.0
0.5
0
***
***
***
**
**
26h
28h
36h
36h
3d
runx1
cmyb
control
kdm6a MO

Figure S4

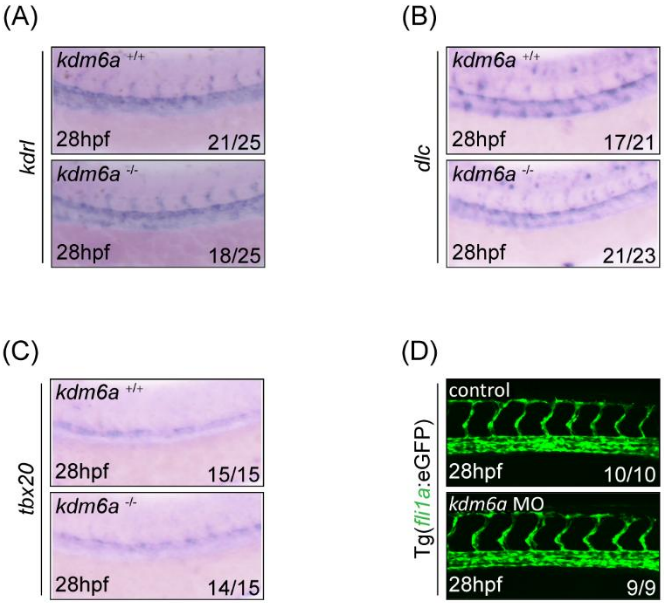
(A)
kdrl
kdm6a +/+
28hpf
21/25
kdm6a -/-
28hpf
18/25
(B)
dlc
kdm6a +/+
28hpf
17/21
kdm6a -/-
28hpf
21/23
(C)
tbx20
kdm6a +/+
28hpf
15/15
kdm6a -/-
28hpf
14/15
(D)
Tg(fli1a:eGFP)
control
28hpf
10/10
kdm6a MO
28hpf
9/9

Figure S5

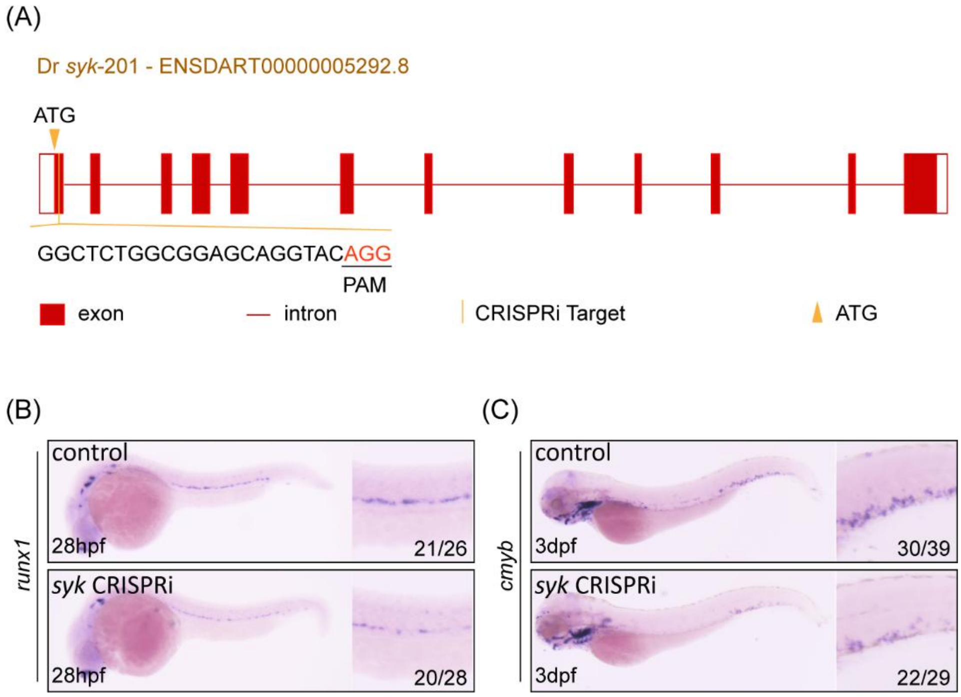

(A)
Dr syk-201 - ENSDART00000005292.8
ATG
GGCTCTGGCGGAGCAGGTACAGG
PAM
exon
intron
CRISPRi Target
ATG
(B)
control
28hpf
21/26
runx1
syk CRISPRi
28hpf
20/28
(C)
control
3dpf
30/39
cmyb
syk CRISPRi
3dpf
22/29

Figure S6

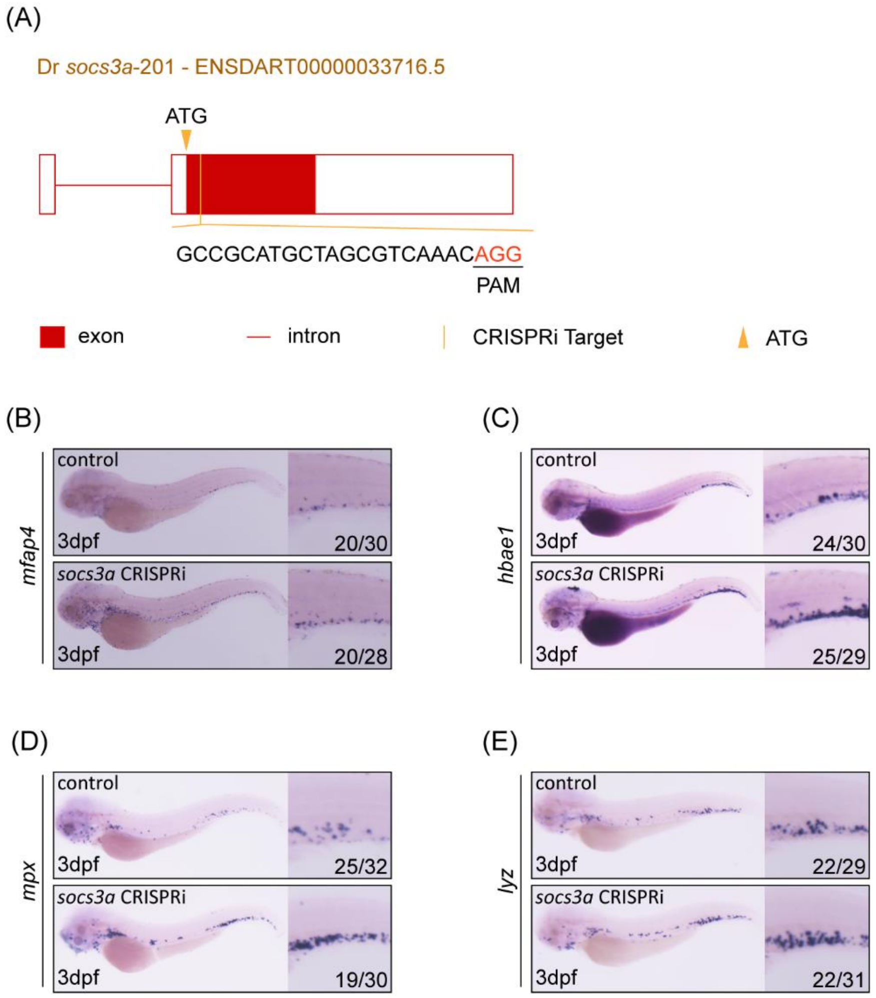
(A)
Dr socs3a-201 - ENSDART00000033716.5
ATG
GCCGCATGCTAGCGTCAAACAGG
PAM
exon
intron
CRISPRi Target
ATG
(B)
mfap4
control
3dpf
20/30
socs3a CRISPRi
3dpf
20/28
(C)
hbae1
control
3dpf
24/30
socs3a CRISPRi
3dpf
25/29
(D)
mpx
control
3dpf
25/32
socs3a CRISPRi
3dpf
19/30
(E)
lyz
control
3dpf
22/29
socs3a CRISPRi
3dpf
22/31

Figure S7

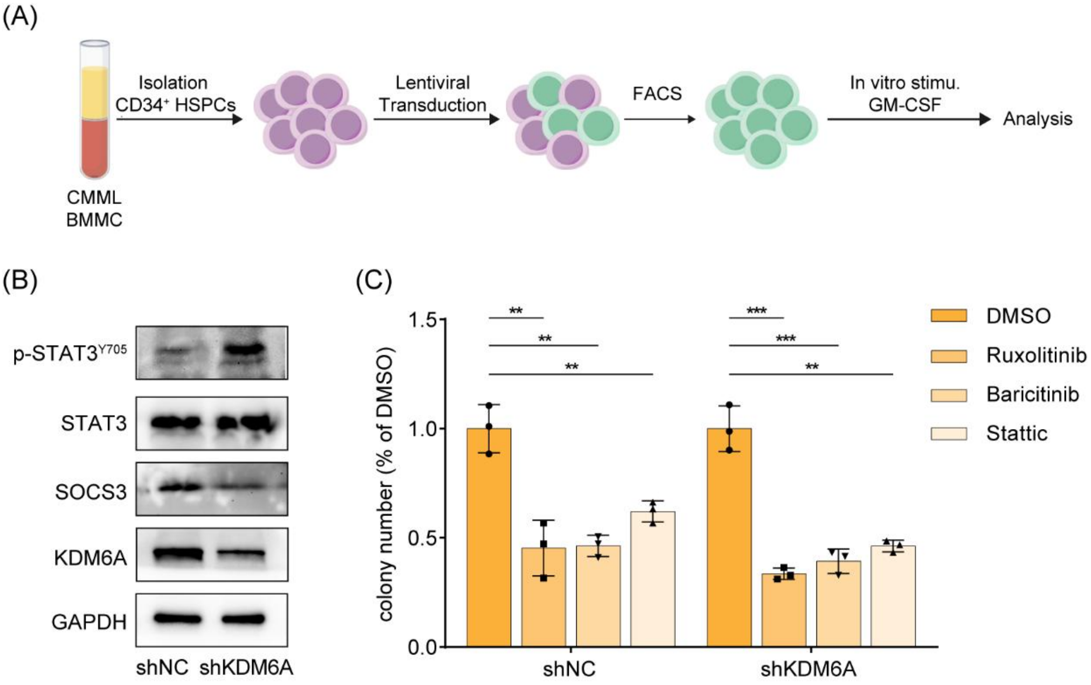
(A)
Isolation
CD34+ HSPCs
Lentiviral
Transduction
FACS
In vitro stimu.
GM-CSF
Analysis
CMML
BMMC
(B)
p-STAT3Y705
STAT3
SOCS3
KDM6A
GAPDH
shNC
shKDM6A
(C)
colony number (% of DMSO)
1.5
1.0
0.5
0.0
**
**
**
***
***
**
shNC
shKDM6A
DMSO
Ruxolitinib
Baricitinib
Stattic

Table S1

real-time PCR primers

Gene	Sequence (5'to3')
zebrafish-*runx1*-qp-FP	ACCGTCTTCACAAACCCTC
zebrafish-*runx1*-qp-RP	CCTGGCTTTACTGCTTCATC
zebrafish-*cmyb*-qp-FP	TTTCTACCGAATCGAACAGATG
zebrafish-*cmyb*-qp-RP	CAATCACCCGTTGGTCTTCT
zebrafish-*syk*-qp-FP	ACACACTATGGCAGCTGGTAG
zebrafish-*syk*-qp-RP	TTCAGCAGAACCCTCAGAATGA
zebrafish-*mpx*-qp-FP	CCTCAACGACAGCACTCTGA
zebrafish-*mpx*-qp-RP	TACTCCAGGTAGGGTTGAGCA
zebrafish-*lyz*-qp-FP	GTGAAAATGGACGGGCTGAA
zebrafish-*lyz*-qp-RP	CTTTGTTTGCGCTGCTCACA
zebrafish-*mfap4*-qp-FP	GTTGGGTTTTTGAGGATTGCCT
zebrafish-*mfap4*-qp-RP	TCAACAGCTCAGTTTATCGTGTGT
zebrafish-*mpeg1*-qp-FP	CACAGAAAACCAGCGCATGAA
zebrafish-*mpeg1*-qp-RP	TTTTGCAAAGCGGGAGTCCA
zebrafish-*hbae1*-qp-FP	CTGAGGCTGTCAGCAAAATCG
zebrafish-*hbae1*-qp-RP	GAACAAAGTGGCCAGAACCAC
zebrafish-*alas2*-qp-FP	TCTATTGCAGGACGCTTGGG
zebrafish-*alas2*-qp-RP	AGACCAGCGCTCCATCTTTC
zebrafish-*cebpd*-qp-FP	TGCGCACCGATTAAGAAGGA
zebrafish-*cebpd*-qp-RP	CGTGTGCATGAAGTTGACGG
zebrafish-*cebpg*-qp-FP	ACAGATCAGAACGGCGTCAG
zebrafish-*cebpg*-qp-RP	GCACTAATTGAGGAACCTGCTG
zebrafish-*cebpz*-qp-FP	CTCTGGGAACTTCAGAAGCTGT
zebrafish-*cebpz*-qp-RP	TCCTGAAGTGGATCTCCGGT
zebrafish-*irf8*-qp-FP	TCCATTTTCAAAGCGTGGGC
zebrafish-*irf8*-qp-RP	AATGTCAAGCTGGGATCGGT
zebrafish-*gata1a*-qp-FP	CTACTCCAGCTCTGAGACTGACC
zebrafish-*gata1a*-qp-RP	TGAGATGAGTAAACTTGGCGAAC
zebrafish-*ccr9a*-qp-FP	ATCATAGAGATCGAGAGGAC
zebrafish-*ccr9a*-qp-RP	CGGTTACATTCATCATGGAT
zebrafish-*socs3a*-qp-FP	AATCAGGCACCAAGAACCTG
zebrafish-*socs3a*-qp-RP	AGTCTCAGCACGCAGTCAAA
zebrafish-*ddx21*-qp-FP	AGCTCACCACCAAACGATGT
zebrafish-*ddx21*-qp-RP	AAGGTGATCCCCGCTTTCTG
zebrafish-*c1qbp*-qp-FP	TGACATTGTCTCGACACCCA
zebrafish-*c1qbp*-qp-RP	GTCTCCATGACCGACCTCGT
zebrafish-*slc25a12*-qp-FP	TTGACTTCGGAGGACATCGT
zebrafish-*slc25a12*-qp-RP	TCCGAACTTGTTCTCCACGC
zebrafish-*18s*-qp-FP	TCGCTAGTTGGCATCGTTTATG
zebrafish-*18s*-qp-RP	CGGAGGTTCGAAGACGATCA
human-*SOCS3*-qp-FP	GGACACTTCGGGAATGCTGA

Table S1 continued

real-time PCR primers

Gene	Sequence (5'to3')
human-*SOCS3*-qp-RP	CTTCCACCTTTCCCAGGCTC
human-*DDX21*-qp-FP	AACTTCTCAAAGGCCGAGGA
human-*DDX21*-qp-RP	TGTCCGTGCCTGTGCAATTA
human-*C1QBP*-qp-FP	ACAGAAGCGAAATTAGTGCGG
human-*C1QBP*-qp-RP	TGTCAGTTCAGGCTCCTGTT
human-*SLC25A12*-qp-FP	ATGGCGGTCAAGGTGCAGA
human-*SLC25A12*-qp-RP	GCTGAACAAAGTCTTCTGGGG
human-*CEBPG*-qp-FP	GCTGCATCTGAGTCTCCTGG
human-*CEBPG*-qp-RP	CAACAACTGATTGCAGGGGC
human-*CEBPZ*-qp-FP	AGTGAGAGCGTTCCAGAACTT
human-*CEBPZ*-qp-RP	TGGCCCTTGAAATGAGCCAG
human-*IRF8*-qp-FP	AAATGCAAACTAGGCGTGGC
human-*IRF8*-qp-RP	GTCCACAGAAGGCTCCTTGA
human-*18S*-qp-FP	GTAACCCGTTGAACCCCATT
human-*18S*-qp-RP	CCATCCAATCGGTAGTAGCG
zebrafish-*syk*-ChIP-1-FP	TGAGCGTACTGCACATCTGTCAT
zebrafish-*syk*-ChIP-1-RP	CTGGATAAGTTGGCGGTTCATTC
zebrafish-*syk*-ChIP-2-FP	CAACACATACACTACGAACACGAG
zebrafish-*syk*-ChIP-2-RP	TGCTAGTAAGTAGATAAGGCGACA
